# Climate‐driven shifts in kelp forest composition reduce carbon sequestration potential

**DOI:** 10.1111/gcb.16299

**Published:** 2022-07-05

**Authors:** Luka Seamus Wright, Albert Pessarrodona, Andy Foggo

**Affiliations:** ^1^ Marine Biology and Ecology Research Centre University of Plymouth Plymouth UK; ^2^ Oceans Institute University of Western Australia Perth Western Australia Australia

**Keywords:** biogeography, carbon budget uncertainty, carbon flux, climate change, C:N, decay, degradation, ecophysiology, erosion, photophysiology

## Abstract

The potential contribution of kelp forests to blue carbon sinks is currently of great interest but interspecific variance has received no attention. In the temperate Northeast Atlantic, kelp forest composition is changing due to climate‐driven poleward range shifts of cold temperate *Laminaria digitata* and *Laminaria hyperborea* and warm temperate *Laminaria ochroleuca*. To understand how this might affect the carbon sequestration potential (CSP) of this ecosystem, we quantified interspecific differences in carbon export and decomposition alongside changes in detrital photosynthesis and biochemistry. We found that while warm temperate kelp exports up to 71% more carbon per plant, it decomposes up to 155% faster than its boreal congeners. Elemental stoichiometry and polyphenolic content cannot fully explain faster carbon turnover, which may be attributable to contrasting tissue toughness or unknown biochemical and structural defenses. Faster decomposition causes the detrital photosynthetic apparatus of *L. ochroleuca* to be overwhelmed 20 days after export and lose integrity after 36 days, while detritus of cold temperate species maintains carbon assimilation. Depending on the photoenvironment, detrital photosynthesis could further exacerbate interspecific differences in decomposition via a potential positive feedback loop. Through compositional change such as the predicted prevalence of *L. ochroleuca*, ocean warming may therefore reduce the CSP of such temperate marine forests.

## Graphical abstract

While warm temperate kelp exports up to 71% more carbon per plant, it decomposes up to 155% faster than its boreal congeners. Faster decomposition causes the detrital photosynthetic apparatus of warm temperate kelp to be overwhelmed 20 days after export, while detritus of cold temperate species maintains carbon assimilation. The predicted prevalence of warm temperate kelp due to ocean warming may reduce the carbon sequestration potential of temperate marine forests.

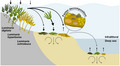

## 1 INTRODUCTION

Over the last decade humans have emitted around 11 billion tons of carbon per year (Canadell et al., 2021). In the Paris Agreement, 193 countries (98% of parties) pledged to reduce this emission and enhance carbon sink capacity. Ocean‐based biological carbon dioxide removal (CDR) is now acknowledged as an integral part of fulfilling this goal (Canadell et al., 2021). Such CDR may be facilitated by marine macrophytes, whose role in carbon sequestration was first recognized four decades ago (Smith, 1981) but has only recently come to mainstream attention as blue carbon (Canadell et al., 2021). Despite the potential involvement of marine vegetated habitats in CDR and their location within national jurisdiction, few ocean‐based nationally determined contributions (NDCs) have been put forward by affluent Annex I parties such as the United Kingdom, United States, and Australia (Gallo et al., 2017), which hold some of the highest blue carbon wealth (Bertram et al., 2021). In part, this may be due to the ongoing debate on the blue carbon status of temperate kelp forests that dominate the coasts of these countries (Krause‐Jensen et al., 2018). Therefore, identifying the magnitude and fate of carbon assimilated by these marine plants is key to our understanding of their carbon sequestration potential (CSP) and their consequent inclusion in blue carbon frameworks (Krause‐Jensen et al., 2018).

**FIGURE 1:**
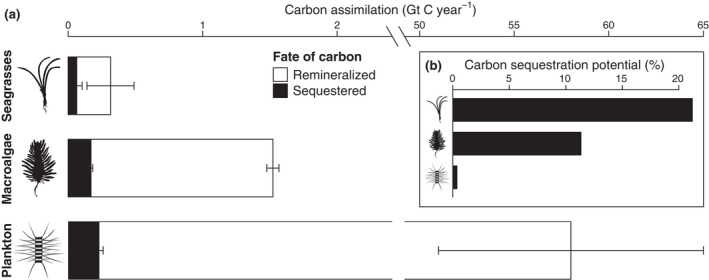
Estimated magnitude and fate of marine autotrophic production. (a) Global marine net carbon assimilation and putative sequestration by fully marine plants (seagrasses, macroalgae, and phytoplankton). A proportion of production is sequestered (black), while the majority is remineralized (white). (b) Percentage of production that is sequestered. Bars and error bars indicate estimated means and putative confidence intervals. For macro‐ and microalgae these are 95% confidence intervals, while error bars for seagrasses represent half range uncertainty (Table S1).

CSP is a function of carbon export and fate (Cebrián et al., 1997; Duarte & Cebrián, 1996) and can be defined as the decline in carbon available for sequestration after export. Carbon export is determined by the magnitude of exported biomass and tissue carbon content (Cebrián et al., 1997; Pedersen et al., 2020; Pessarrodona et al., 2018, 2019), while variance in carbon fate is attributable to differential remineralization. The two processes that constitute remineralization are consumption by detritivores and degradation by microbial saprotrophs (Cebrián et al., 1997; Duarte & Cebrián, 1996), hereafter collectively referred to as decomposition. Marine plants that have higher carbon‐nutrient ratios (Enríquez et al., 1993), slower growth (Cebrián & Duarte, 1995), more refractory compounds (Trevathan‐Tackett et al., 2015) and more polyphenols (Amsler, 2008) tend to decompose more slowly. Therefore, macroalgae generally decompose slower than phytoplankton and faster than seagrasses (Duarte & Cebrián, 1996), endowing them with an intermediate relative CSP (Figure 1b). Nonetheless, they are estimated to cover 9.12–33.31 times more area than seagrasses globally (Duarte et al., 2013, 2022). Hence their absolute CSP (Krause‐Jensen & Duarte, 2016) is thought to be 158% greater than that of seagrasses and only 25% smaller than that of phytoplankton (Figure 1a). Although this estimate is therefore logically robust, uncertainty surrounding macroalgal carbon sequestration remains substantial (Queirós et al., 2019).

Variance in carbon export, decomposition and burial is recognized to bring about large interspecific differences in the CSP of seagrasses (Cebrián et al., 1997; Lavery et al., 2013) and mangroves (Atwood et al., 2017; Li et al., 2018). However, a similar interspecific comparison is lacking for macroalgae, despite two additional major sources of variability in this group. Detrital floating time and transport distance of macroalgae range from a few seconds (Wernberg & Filbee‐Dexter, 2018) and meters (Filbee‐Dexter et al., 2018) to thousands of hours (Tala et al., 2019) and kilometers (Fraser et al., 2018), while they are more consistent for the detritus of marine angiosperms (Harwell & Orth, 2002; Perry et al., 2018). The current global estimate of macroalgal CSP (Krause‐Jensen & Duarte, 2016) was calculated using between 1 and 20 distinct genera for each parameter (Table S2) and hence does not reflect this variability. Importantly, data on kelp forests, which constitute the largest and most productive macroalgal biome on the planet (Duarte et al., 2022), are underrepresented (Table S2). Considering that these estimates have nonetheless already been used to calculate kelp CSP (Filbee‐Dexter & Wernberg, 2020), we argue that a better understanding of interspecific differences is urgently needed to reliably resolve the contribution of macroalgae to blue carbon sequestration.

**FIGURE 2:**
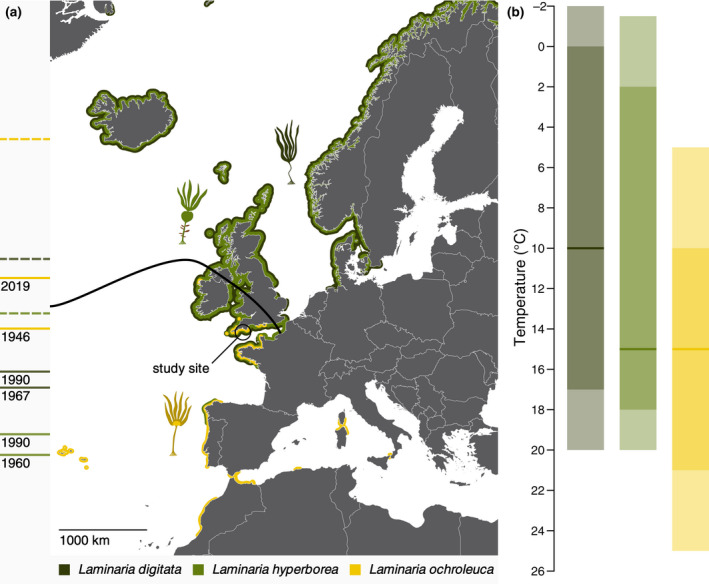
Biogeography of Northeast Atlantic *Laminaria* species. (a) Current approximate species distributions are shown as coloured coastlines. Past and future (SRES A2 or RCP8.5) trailing (*Laminaria digitata* and *Laminaria hyperborea*) or leading (*Laminaria ochroleuca*) range edges are indicated by solid and dashed lines in the left margin, respectively (Table S3). Kelp icons denote approximate range centres. The black line shows the northern biogeographic boundary of warm temperate kelps (Table S3). The map is based on the coordinate reference system WGS 84, rendered according to the Mercator projection and oriented north. (b) Underlying these species distributions are the species‐specific temperature optima (lines) and tolerances (shaded areas) of sporophyte growth (light) and gametophyte fertility (dark; Table S4). RCP, representative concentration pathway.

Here we show that interspecific variance in CSP within a single macroalgal genus is large, using kelp forests in the southern United Kingdom as a model system (Figure 2a). These forests have experienced rapid, climate‐driven range shifts in species composition over the last century, with increased dominance of a warm temperate kelp (Parke, 1948; Pessarrodona et al., 2019; Smale et al., 2015) and further changes are expected in the near future (Figure 2a). Additionally, our study location is the only locality worldwide where macroalgal carbon burial in coastal sediments has been empirically documented in situ (Queirós et al., 2019). We first outline differences in carbon export and decomposition speed of *Laminaria* species and investigate potential mechanistic drivers. Based on these parameters we then estimate how CSP differs between species and thus relates to kelp forest composition. The additional dimension of biogeographic shifts and subsequent compositional change gave us the opportunity to also model the effect of ocean warming on the densities of kelps with different thermal tolerances (Figure 2b), based on historical and future (representative concentration pathway 2.6 [RCP2.6], RCP6.0, and RCP8.5) sea surface temperature. We finally explore the photophysiology and biochemistry of decomposing detritus to gain further insights into potential feedback loops.

## 2 METHODS

### 2.1 Model system

Northeast Atlantic temperate marine forests are mostly dominated by three species of the genus *Laminaria* (Lüning, 1990). Based on their distribution (Figure 2a) and underlying thermal tolerances (Figure 2b), two of these species are classed as cold temperate (*Laminaria digitata* and *L. hyperborea*) and one as warm temperate (*Laminaria ochroleuca*; Lüning, 1990). In addition to their differential thermal tolerance, these species have contrasting growth and senescence phenologies (Hereward et al., 2018; Pessarrodona et al., 2019). Furthermore, the boreal species grow vertically separated in the shallow infralittoral, with *L. digitata* occupying the upper zone adjacent to the eulittoral fringe (Lüning, 1990). In the geographic region of overlap between northern Spain and the southern United Kingdom (Figure 2a), their warm temperate congener coexists with *L. hyperborea* in mixed stands, albeit inhabiting somewhat deeper substrata (L. S. Wright, personal observation). Its depth affinity is evidenced by the ability of *L. ochroleuca* to grow in the Mediterranean circalittoral (Bartsch et al., 2008) and inability to occupy the *L. digitata* band due to physiological stress (King et al., 2018).

With the aim of studying differences in the carbon cycle of these *Laminaria* species, we collected a comprehensive data set on plant density and mass (standing stock), particulate carbon export, detrital primary production and decomposition in Plymouth Sound, UK (Figure S1), which is situated near the the northern biogeographic boundary of warm temperate kelps (Figure 2a). Our studies were conducted along the full vertical extent of the ~100‐m^2^ West Hoe kelp forest (50.363629°N, 4.144978°W), while we also made use of the infralittoral sedimentary sites off Drake's Island (50.353328°N, 4.150224°W) and Jennycliff (50.343059°N, 4.131762°W) for decomposition experiments on alternative, deeper substrata (Figure S1).

### 2.2 Empirical data

#### 2.2.1 Sporophyte density and mass

The sporophyte density of each *Laminaria* species at West Hoe was measured during 5 months of the year across all seasons between January 2016 and March 2017. Mature plants (>15 cm) were counted by haphazardly placing 1‐m^2^ quadrats in the lower eulittoral zone, where *L. digitata* occurs most abundantly, and in the upper infralittoral zone, where *L. hyperborea* and *L. ochroleuca* are prevalent, during low spring tides. Mature sporophytes were also harvested and their wet mass recorded to the closest gram.

#### 2.2.2 Carbon export

To measure species‐specific particulate carbon export, we quantified the release of detritus via distal lamina erosion at West Hoe between March 2016 and February 2017. We used erosion as a conservative measure of the total carbon exported from kelp forests, which includes carbon exported as entire dislodged plants (de Bettignies et al., 2013; Pedersen et al., 2020; Pessarrodona et al., 2018) as well as dissolved carbon export (Abdullah & Fredriksen, 2004; Blain et al., 2021; Weigel & Pfister, 2021). Erosion was quantified using the hole punch method (Krumhansl & Scheibling, 2011a; Tala & Edding, 2005). Each month, ten plants of each species were tagged and uniquely labelled during low spring tides. Three holes were punched above the stipe‐lamina connection of every sporophyte, two on the central digit and another one on an outer digit. This was done to capture inter‐ and intra‐digit variability in growth, which is known to peak between 2.5 and 15 cm from the stipe‐lamina transition (Kain, 1976). Initial hole position (*H*_I_) and the initial length of each punched digit (*L*_I_) were recorded. After a month, tagged plants were collected and returned to the laboratory, where the final digit lengths (*L*_F_) and final hole positions (*H*_F_) for each plant were measured. The distal lamina loss for each plant (*E*_L_, cm plant^−1^ month^−1^) was calculated as

$$E_{L}=\;L_{I}+G–L_{F,}$$

where *G*, the growth of each digit (cm month^−1^), is *H*_F_ – *H*_I_ (data not shown). Since *L*_F_ ≤ *L*_I_ + *G*, *E*_L_ yields the digit length lost to distal erosion. For each plant, *E*_L_ was averaged across the central and outer digits. To convert the loss of tissue length (cm) to biomass (g), three 5‐cm segments from the most distal part of each retrieved lamina were cut and their wet mass recorded. We determined the relationship between wet and dry mass by drying the outermost segment at 60°C for 48 h. Wet mass consistently correlated well with dry mass (*R* ^2^ ≥ .89). We then estimated the dry mass of the rest of the 5‐cm segments for each plant using this wet to dry mass ratio. Dry mass per unit length was averaged between the three segments to give the distal lamina dry mass to length ratio (ML_R_, g cm^−1^). The daily erosion rate (*E*_M_, g plant^−1^ day^−1^) was calculated as

$$E_{M}=\frac{E_{L}\times {\mathrm{ML}}_{R}}{∆t},$$

where *E*_L_ is lamina length loss (cm plant^−1^ month^−1^) and Δ*t* is the number of elapsed days (days month^−1^). Finally, these biomass erosion rates were converted to carbon export (g C plant^−1^ day^−1^) using site‐ and species‐specific carbon content (%) obtained from sampling three mature sporophytes approximately every 2 months. Kelp tissue from each sampled individual was lyophilized (Lablyo, Frozen in Time Ltd), ground and its carbon content quantified using an elemental analyser (CHN analyser, EA1110; CE Instruments Ltd; see methods Section 2.2.5 for details).

#### 2.2.3 Decomposition

In situ decomposition rates were derived from two litterbag experiments, which were independently conducted in 2016 and 2019 respectively. In 2016, lamina material from each *Laminaria* species was collected in March and cut into ~5 × 15‐cm strips. A total of 105 ± 8 g of fresh kelp strips was then sealed within eight mesh bags (2‐ and 20‐mm mesh ø) per species, which were then deployed at 4 m below lowest astronomical tide on a sandy seabed adjacent to kelp forests at Drake's Island and Jennycliff. Litter bags were attached to a long rope, positioned approximately 1 m apart from one another, and secured to the seabed with anchor weights. Upon retrieval after 40–41 days, a fine mesh bag (1‐μm ø) was placed over each litter bag to retain all kelp material and detritivores, before detaching the bag from the rope. In the laboratory, the contents of the bags were carefully removed and washed through a 1‐mm sieve. Remaining kelp tissue was weighed.

In 2019, nine mature sporophytes of each species were collected at West Hoe on May 17 and four fronds removed from each in situ. The first of these was placed in a cooler for transport to the laboratory, whereas the other three were trimmed to weigh 20 ± 1 g. Frond samples were taken from the central part of the current year's lamina growth, avoiding the meristematic basal and eroding distal regions. For each species, one frond sample from each sporophyte was placed within each of three rectangular, galvanized steel mesh enclosures (62.4 × 34.4 cm, 13‐mm mesh ø). The nine mesh cages were then chained together, closed with cable ties and deployed at 2 m below lowest astronomical tide within the kelp forest at West Hoe. A temperature and light logger (HOBO Pendant®) was secured to one end of the chain facing the surface and the chain was deployed parallel to the shoreline to control for depth. Samples were retrieved on May 30, June 11, and June 18 (i.e., after 13, 25, and 32 days). On each retrieval date, three randomly selected frond samples were removed from each litter bag and weighed using an analytical balance (±1 mg, Fisherbrand™️ Precision Series; Fisher Scientific). Two subsamples (1.5–2 g) were then refrigerated at ~3°C to retain cellular function for subsequent photophysiological measurements, while the rest of the sample was frozen at –20°C for elemental and phenolic analyses.

Using mass data from both experiments, decomposition (*D*, day^−1^) was calculated as

$$D=\frac{M_{0}–M_{1}}{M_{0}\times ∆t},$$

where *M*_0_ is the initial sample mass, *M*_1_ is the retrieved sample mass (g), and Δ*t* is the time period (days).

#### 2.2.4 Detrital primary production and respiration

Gross primary production (GPP) is true photosynthesis minus photorespiration, while net primary production (NPP) further accounts for respiration (R; Wohlfahrt & Gu, 2015). According to the definition used here, NPP attains negative values when photosynthetic supply cannot cover respiratory demand and stored carbon (i.e., old production) is respired in addition to new production (Roxburgh et al., 2005).

NPP and R of refrigerated duplicate subsamples (methods Section 2.2.3) from West Hoe were measured via closed bottle respirometry within five (more often two) days of collection. These measurements were performed by quantifying light and dark oxygen (O_2_) evolution in 130 ± 5‐ml glass incubation jars (cf. Lüning, 1979). Sample mass was recorded as buoyant rather than blotted wet mass to keep the microbial biofilm intact and later converted. Incubations were exposed to 50.4 μmol photons m^−2^ s^−1^. This light treatment is within the saturation range for *Laminaria* species (Bartsch et al., 2008) and representative of a previously measured mean *Laminaria* forest photoenvironment at 2 m depth in spring (60.7 μmol photons m^−2^ s^−1^, Lüning & Dring, 1979). Each set of measurements was accompanied by a 270 ± 5‐ml blank incubation. All incubation jars were fitted with 5‐mm diameter planar oxygen‐sensitive spots (PreSens) and magnetic stir bars (Fisherbrand™️; Fisher Scientific) and placed on a magnetic stirrer (MIX 15 eco; 2mag AG), set at 350 rpm. Measurements were taken with a fiber optic O_2_ meter (Fibox 4 trace; PreSens) after 10 and 30 min in a 20°C controlled temperature room. Any fluctuation in temperature was accounted for by the O_2_ meter's temperature probe, which was placed in a water bath alongside the stirrer. Seawater salinity (33–35‰) was measured prior to incubation and the O_2_ meter's settings were modified accordingly. NPP (μmol O_2_ g^−1^ min^−1^), R (μmol O_2_ g^−1^ min^−1^), and GPP (μmol O_2_ g^−1^ min^−1^) were subsequently calculated as

$$\mathrm{NPP}=\frac{\left({c_{S}}_{1}–{c_{S}}_{0}\right)\times V_{S}–\left({c_{B}}_{1}–{c_{B}}_{0}\right)\times V_{B}\;}{M\times ∆t},$$

$$R=\frac{\left({c_{S}}_{0}–{c_{S}}_{1}\right)\times V_{S}–\left({c_{B}}_{0}–{c_{B}}_{1}\right)\times V_{B}\;}{M\times ∆t},$$

GPP=NPP+R,

where *c* is the molar O_2_ concentration (μmol O_2_ L^−1^), *V* is the incubation volume (L), *M* is the sample wet mass (g) and Δ*t* is the time window (min). _S_ refers to incubations containing a sample, while _B_ refers to blank incubations. Subscript numbers represent the time of measurement. All values were averaged across the 10‐ and 20‐min incubation periods for each sample and converted to μmol O_2_ g^−1^ h^−1^. Net carbon assimilation per g of dry mass (*CA*, g C g^−1^ h^−1^) was then calculated from NPP (μmol O_2_ g^−1^ h^−1^) as

$$\mathrm{CA}=\frac{\mathrm{NPP}\times 10^{–6}\times 12.0107\;}{M_{R}},$$

where 10^−6^ is the conversion factor from μmol to mol, 12.0107 is the atomic mass of carbon and *M*_R_ is the dry mass to wet mass ratio measured at West Hoe in May 2016. Gross CA was calculated from GPP in the same way. This calculation assumes a photosynthetic quotient (PQ) and respiratory quotient of 1. Although PQ changes with depth (Miller & Dunton, 2007) and light exposure (Miller et al., 2009) in *L. hyperborea* and varies interspecifically in brown algae (Thomas & Wiencke, 1991), an extensive literature search and correspondence with various European phycologists revealed no usable PQs for our interspecific comparison.

On the first day of sample retrieval (30 May 2019), air temperature (22.9°C) and photon fluence rate (2618 μmol photons m^−2^ s^−1^) were extremely high. At 971 μmol photons m^−2^ s^−1^, well below the observed irradiance on that day, photoinhibition was previously shown to reduce in situ O_2_ production of *L. digitata* (Delebecq et al., 2011). After photoinhibition was confirmed during the data exploration stage, primary production and respiration data from this day were removed from the statistical analysis.

#### 2.2.5 Elemental stoichiometry

Frozen subsamples were lyophilized (Lablyo, Frozen in Time Ltd) and subsequently ground to 250‐μm powder. Powder samples of ~2 mg (±0.01 mg, AT201; Mettler Toledo) were then sealed in 6 × 4‐mm tin capsules (OEA Laboratories Ltd) and combusted in an elemental analyser CHN analyser, EA1110; CE Instruments Ltd) to measure their carbon and nitrogen content (%). Acetanilide (C_8_H_9_NO; OEA Laboratories Ltd) was chosen as the analytical standard because of its high carbon (71.09%) and low nitrogen (10.36%) content.

#### 2.2.6 Phenolic content

Total soluble polyphenolic content was measured using a high‐throughput 96‐well microplate Folin‐Ciocalteu colorimetric assay (cf. Hargrave et al., 2017). 50 mg of ground, freeze‐dried kelp was added to 500 μl of 100% methanol and vortexed (Whirlimixer; Fisons) for ~10 s in a 1.5‐ml microtube. The solute was left to extract for 1 h, after which the 10% (w/v) solution was vortexed again and then centrifuged at 14,000 × *g* for 5 min. The supernatant was transferred to another microtube and immediately stored at −20°C. Phloroglucinol (PG; Sigma‐Aldrich), used as the phenol standard, was dissolved in 100% methanol at concentrations of 0.05, 0.1, 0.25, 0.5, 0.75 and 0.1 mg ml^−1^. In a 96‐well microplate, 10 μl of each PG solution was added to 100 μl of 10% (diluted with distilled water) Folin‐Ciocalteu reagent (Sigma‐Aldrich) and, after a 5‐min reaction period, 90 μl of 1 m Na_2_CO_3_ solution was added last. To produce the standard curve, absorbance of each PG concentration was measured at 765 nm using a microplate reader (FLUOstar® Omega; BMG Labtech Ltd). After centrifugation at 14,000 × *g* for 1 min to eliminate remaining particulates, sample extracts were prepared and their absorbance recorded as described above. All measurements were taken in triplicates and converted to PG equivalents using the standard curve.

#### 2.2.7 Grazing pressure

Grazing impact by dominant macrodetritivores such as *Steromphala cineraria* (de Bettignies, Dauby, Lepoint, et al., 2020; Hargrave et al., 2017; Pessarrodona et al., 2019; Smale et al., 2015) and *Patella pellucida* (de Bettignies, Dauby, Lepoint, et al., 2020; Hargrave et al., 2017; Hereward et al., 2018; Pessarrodona et al., 2019; Smale et al., 2015) was estimated by measuring the area of excavation and perforation scars via image analysis (cf. Hereward et al., 2018) after in situ decomposition. Excavation here refers to a paling of the lamina surface that is associated with excavation by algivorous gastropods (Krumhansl & Scheibling, 2011b) but may also be caused by saprotrophic microbes. Perforations are defined as holes in the lamina that are most likely caused by gastropod radulae during prolonged grazing (Krumhansl & Scheibling, 2011b). Samples, retrieved after 32 days (June 18, 2019), were photographed next to a ruler (±1 mm) under identical lighting. Images were then analysed with Fiji (ImageJ v2.0.0‐rc‐69/1.52p; Schindelin et al., 2012). The surface areas of holes and discolored tissue were measured manually, while total surface area was measured by making the image binary and highlighting the sample edges. The proportional areas of excavated (*E*) and perforated (*P*) lamina were calculated as

$$E=\;\frac{A_{E}}{A_{T}–A_{P}},$$

$$P=\;\frac{A_{P}}{A_{T}},$$

where *A*_E_ is the excavated area, *A*_T_ is the total area and *A*_P_ is the perforated area.

### 2.3 Estimation

#### 2.3.1 Present areal carbon export and CSP

Species‐specific CSP was estimated using empirical sporophyte density, carbon export and decomposition data (methods Sections 2.2.1, 2.2.3). The CSP estimation was carried out in R v4.1.2 (R Core Team, 2022) within the integrated development environment RStudio v2021.09.2 (RStudio Team, 2022) and can be accessed at github.com/lukaseamus/CSP/tree/main/Sequestration.

The kelp forest at West Hoe forms a ~20‐m band, the upper ~4 m of which are occupied only by *L. digitata*, whereas the lower ~16 m consist of a mixed stand of *L. hyperborea* and *L. ochroleuca* (L. S. Wright, personal observation). To account for this vertical distribution, sporophyte density (plants m^−2^) for each species was multiplied by the relative space it occupies in the kelp forest (0.2 in the case of *L. digitata* and 0.8 in the case of *L. hyperborea* and *L. ochroleuca*). Seasonal and annual carbon export were obtained by multiplying daily carbon export (g C plant^−1^ day^−1^) for each month by the number of days in that month, then calculating the mean monthly carbon export and finally summing those means across season or year. According to the variance sum law, standard errors of the annual means (*SE*_S_) were estimated as

$${\mathrm{SE}}_{S}=\sqrt{{\mathrm{SE}}_{1}^{2}+{\mathrm{SE}}_{2}^{2}+\ldots +{\mathrm{SE}}_{12}^{2}},$$

where *SE*_1–12_ are the standard errors of individual months. See github.com/lukaseamus/CSP/tree/main/Export for details. The change in present seasonal and annual *CSP* (g C m^−2^ season^−1^ or g C m^−2^ year^−1^) with detrital age estimated for each species was

$$\mathrm{CSP}=N\times \mathrm{CE}\times \left(1–t\times D\right),$$

where *N* is the seasonal or annual mean number of sporophytes (plants m^−2^), CE is the seasonal or annual carbon export (g C plant^−1^ season^−1^ or g C plant^−1^ year^−1^), *t* is the detrital age (days) and *D* is decomposition (day^−1^) in spring and early summer (methods Section 2.2.3). According to the rules of estimating variance around the product of two means (Buonaccorsi & Liebhold, 1988), the seasonal and annual 95% confidence intervals (CI) around CSP were estimated for each species as

$$\mathrm{CI}=\mathrm{CSP}\pm z\times \sqrt{{\mathrm{SE}}_{N}^{2}\times {\mathrm{SE}}_{\mathrm{CE}}^{2}+{\mathrm{SE}}_{N}^{2}\times {\bar{X}}_{\mathrm{CE}}^{2}+{\mathrm{SE}}_{\mathrm{CE}}^{2}\times {\bar{X}}_{N}^{2}},$$

where *z* is the 97.5 percentile point of the standard normal distribution, ${\bar{X}}_{N}$ and *SE*_N_ are the seasonal or annual means and standard errors of sporophyte density (plants m^−2^) and ${\bar{X}}_{\mathrm{CE}}$ and *SE*_CE_ are the seasonal or annual means and standard errors of carbon export (g C plant^−1^ season^−1^ or g C plant^−1^ year^−1^). The same equation was used to estimate 95% confidence intervals around estimates of annual areal carbon export (g C m^−2^ year^−1^).

#### 2.3.2 Areal carbon export and CSP through time

To test the effect of past and future ocean temperature on CSP, sporophyte densities were modelled according to species‐specific temperature tolerances (Figure 2b; Table S4), historical sea surface temperature data at 1° spatial resolution (Rayner, 2003) and RCP temperature predictions at 5‐arcmin spatial resolution (Assis et al., 2018) for the region around West Hoe (50.363629°N, 4.144978°W). Minimum (February), mean (annual average), and maximum (August) temperature data were extracted from both data sets with the R package raster v3.4‐5 (Hijmans, 2020) and a trendline was fit using locally estimated scatterplot smoothing (polynomial regression) with smoothing parameter *ɑ* = 1 (Figure S5).

In the region of latitudinal range overlap (Figure 2a), warm temperate *L. ochroleuca* is currently limited by minimum temperatures while cold temperate *L. digitata* and *L. hyperborea* are limited by maximum temperatures (van den Hoek, 1982). It is likely that the limiting factor at the leading range edge is gametophyte fertility, since the spores which gametophytes develop from are the only mobile life stage of *Laminaria* species and gametophytes are therefore the first to arrive in a new locality (Bartsch et al., 2008). This is supported by the arrival of *L. ochroleuca* in Plymouth Sound in 1946 (Parke, 1948), when maximum sea surface temperatures were approximately 9.53°C, close to the lowest tolerance of 10°C for gametophyte fertility (Table S4). Conversely, trailing edge populations are likely limited by sporophyte growth. While gametophytes are short‐lived even under artificially constant dark conditions, *Laminaria* sporophytes are perennial and can grow up to 18 years old (Bartsch et al., 2008). Therefore *Laminaria* sporophytes may persist for several years with potentially stunted growth after temperatures become suboptimal for reproduction. This is evidenced by the slower retraction of trailing edge populations of benthic macroalgae than their leading edge populations expand (García Molinos et al., 2017). Therefore, using the estimated maximum (*T*_max_) and minimum (*T*_min_) temperature trends, plant densities for cold temperate (*N*_C_) and warm temperate (*N*_W_) species were modelled through time as

$$N_{C}=\frac{N\times \left(T_{U}–T_{\max}\right)\;}{T_{U}–T_{\max2016}},$$

$$N_{W}=\frac{N\times \left(T_{\min}–T_{L}\right)\;}{T_{\min2016}–T_{L}},$$

where *N* is the mean number of plants (m^−2^) in 2016 (methods Section 2.2.1), *T*_U_ is the upper temperature limit (i.e., just above the highest tolerated temperature) for cold temperate sporophyte growth (cf. Figure 2b), *T*_L_ is the lower temperature threshold for warm temperate gametophyte fertility (cf. Figure 2b, note that this was adjusted from 10 to 9.53°C because Parke (1948) found that *L. ochroleuca* established a population at this sea surface temperature), *T*_min2016_ and *T*_max2016_ are the minimum and maximum sea surface temperatures for 2016, the year for which plant density measurements are available (methods Section 2.2.1). Rather than mathematically defining optima, the above equations focus on temperature extremes (i.e., tails of the unimodal curve) for which the polynomial equation is simplified here as an ordinary linear equation. Hence, as estimated Δ*T* (*T*_U_ – *T*_max_ or *T*_min_ – *T*_L_) increases or decreases relative to Δ*T*_2016_ (*T*_U_ – *T*_max2016_ or *T*_min2016_ – *T*_L_), the proportion by which *N* is multiplied linearly increases or decreases respectively. That way, as the temperature threshold is neared (i.e. Δ*T* decreases), density decreases linearly.

Importantly, *L. ochroleuca* cannot occupy the vertical zone of *L. digitata* because it cannot tolerate emersion, mostly due to cold stress rather than desiccation (King et al., 2018). Therefore, the plant density of this warm temperate species was limited to the total observed sporophyte density (*L. hyperborea* + *L. ochroleuca*) in the lower ~16‐m band of the kelp forest in 2016. Using the modelled plant densities (*N*_M_, m^−2^), long‐term trends in annual carbon export (*CE*_T_, g C m^−2^ year^−1^) and CSP (*CSP*_T_, g C m^−2^ year^−1^) were estimated as

$${\mathrm{CE}}_{T}=N_{M}\times \mathrm{CE},$$

$${\mathrm{CSP}}_{T}=N_{M}\times \mathrm{CE}\times \left(1–t\times D\right),$$

where CE is annual carbon export (g C plant^−1^ year^−1^), *t* is the detrital age (days) at which sequestration is assumed and *D* is decomposition (day^−1^). Here we used *t* = 50 days to illustrate a scenario where all species would contribute to overall CSP. Trends in annual CE and CSP for the entire *Laminaria* forest were derived by summing CE_T_ and CSP_T_ across species. The variance sum law was again applied to estimate 95% confidence intervals (methods Section 2.3.1).

#### 2.3.3 Cumulative detrital carbon assimilation potential

Detritus of *Laminaria* species maintains primary production for months (de Bettignies, Dauby, Thomas, et al., 2020; Frontier, de Bettignies, et al., 2021; Frontier, Mulas, et al., 2021). However, it also sinks immediately due to lack of pneumatocysts or porous tissue, as was previously observed for *L. hyperborea* (Wernberg & Filbee‐Dexter, 2018). Nonetheless, kelp detritus may assimilate significant amounts of carbon while resting on shallow seabed as evidenced by the growth of some *Laminaria* detritus (de Bettignies, Dauby, Thomas, et al., 2020; Frontier, Mulas, et al., 2021; Pedersen et al., 2021). The reason may be periodically high photon flux densities (e.g. 142.7 μmol photons m^−2^ s^−1^ at 2 m depth in July, Lüning & Dring, 1979) that cause photosynthesis to mask decomposition. These findings suggest that macroalgal detritus travelling through illuminated zones can resist carbon loss, although experiments have shown that photophysiological responses differ interspecifically (Frontier, de Bettignies, et al., 2021; Frontier, Mulas, et al., 2021). During our second in situ decomposition experiment (methods Section 2.2.3), light was very limited due to shading by the kelp canopy and turbidity (daytime mean = 0.54 μmol photons m^−2^ s^−1^, overall mean = 0.36 μmol photons m^−2^ s^−1^). This invited an exploration of how our detritus might have assimilated carbon had there been different photon flux densities.

To estimate how net carbon assimilation may change throughout the detrital phase, we made use of our empirical gross carbon assimilation data (methods Section 2.2.4), the mentioned low‐light regime during the decomposition experiment, seasonal and annual average daylight hours for the Plymouth Sound region, depth profiles of photosynthetically active radiation (PAR) for the Plymouth Sound region between 2009 and 2020 (Western Channel Observatory, 2021), the photosynthesis‐irradiance relationship for *L. hyperborea* (Duarte, Ramos, et al., 2013) along with the location and depth of the local kelp carbon sink (Queirós et al., 2019). Because respiration is assumed to stay constant under changing light (GPP is calculated as NPP + R on these grounds), we were able to estimate the potential for detrital net carbon assimilation under various light regimes.

First, the seasonal and annual excess (in a brighter photoenvironment) gross carbon assimilation as a proportion of laboratory GPP needs to be determined. In the following equation the term left of the multiplication sign calculates the proportion of laboratory GPP that remains after accounting for in situ GPP during our decomposition experiment. The right term signifies the proportion of laboratory GPP at various stages in the detrital phase. When multiplied, the terms thus yield a function that gives the proportion by which laboratory GPP must be adjusted (*P*_GPP_):

$$P_{\mathrm{GPP}}=\left(1–\frac{\mathrm{PE}\left({\mathrm{PAR}}_{F}\right)}{\mathrm{PE}\left({\mathrm{PAR}}_{L}\right)}\right)\times \frac{\mathrm{PE}\left({\mathrm{PAR}}_{D}\left(t\times v÷\sqrt{m^{2}+1}\times m\right)\right)}{\mathrm{PE}\left({\mathrm{PAR}}_{L}\right)},$$

where PE is the photosynthesis‐irradiance function $y=1.8\times \tan h\left(\frac{0.006x}{1.8}\right)$, PAR_F_ and PAR_L_ are the mean daytime photon flux density during the field experiment (0.54 μmol photons m^−2^ s^−1^) and the laboratory irradiance (50.4 μmol photons m^−2^ s^−1^), PAR_D_ is an exponential decay function of seasonal or annual light attenuation with depth (e.g. the annual equation is $y=e^{–0.15x+5.05}$), *t* is the detrital age (days), *v* is the minimal detrital velocity (m day^−1^), estimated by trigonometry from the depth of the local carbon sink and its distance from the nearest kelp forest, assuming a detrital travel time of 50 days (methods Section 2.3.2), and *m* is the seabed slope, estimated by dividing the depth of the local carbon sink by its distance from the West Hoe kelp forest.

Second, the change in seasonal or annual cumulative net carbon assimilation (*CA*, g C m^−2^ season^−1^ or g C m^−2^ year^−1^) with detrital age was estimated as

$$\;\mathrm{CA}=\sum_{k=1}^{t}N\times \mathrm{BE}\times D_{\mathrm{CA}}\left(k\right)\times h\times \mathrm{GPP}\left(k\right)\times P_{\mathrm{GPP}}\left(k\right),$$

where *t* is the detrital age (days), *N* is the seasonal or annual sporophyte density (plants m^−2^), BE is the seasonal or annual dry biomass export (g plant^−1^ season^−1^ or g plant^−1^ year^−1^), *D*_CA_ is a special case of linear decomposition that never attains negative values (i.e. *D*_CA_ = 1 – *t* × *D* where *t* × *D* ≤ 1; methods Section 2.3.1), *h* is the seasonal or annual daylight time (h) and GPP is the species‐specific gross carbon assimilation (g C g^−1^ h^−1^). Note that this estimate of detrital carbon assimilation is conservative, since it assumes that excess production in the new light milieu cannot photosynthesise: $N\times \mathrm{BE}\times D_{\mathrm{CA}}\left(k\right)$ always gives the remaining biomass at each *k* irrespective of elevated production. As such CA is the carbon pool that a shrinking amount of detritus can produce as it travels to the sink. The 95% confidence intervals for CA are assumed to be the same as those of the other estimates (methods Section 2.3.1).

### 2.4 Data analysis and visualisation

Data analysis and visualisation were performed in R v4.1.2 (R Core Team, 2022) within the integrated development environment RStudio v2021.09.2 (RStudio Team, 2022). The output of all analyses is listed in Table S6.

Prior to analysis, data were explored using standard visualisation techniques (Zuur et al., 2009). For seven response variables, all assumptions were met and standard linear models were built (Table S6). If the data distribution violated the assumption of normality, alternative distributions were explored with fitdistrplus v1.1‐6 (Delignette‐Muller & Dutang, 2015). In four cases, a gamma generalised linear model with a logarithmic link function fit the data best (Table S6). In the case that the assumption of homogeneity was not met, variance was modelled as a function of explanatory variables (Zuur et al., 2009) with generalised least squares in nlme v3.1‐153 (Pinheiro et al., 2021). This model type fit the data best in six cases (Table S6). For every response variable, the potential influence of individual mesh bags was checked by building linear mixed effects models in lme4 v1.1‐27.1 (Bates et al., 2015) or nlme with mesh bag identity as a random intercept and slope. These models were then tested against fixed effects models and in a single case mesh bag identity was determined to explain some of the variation in the response variable (Table S6). Type II or III omnibus hypothesis tests were performed with car v3.0‐12 (Fox & Weisberg, 2019).

The package ggplot2 v3.3.5 (Wickham, 2016) was used for data visualisation. In the specific cases of decomposition and monthly variation in daily carbon export, ggplot2 was augmented with the probability density and x‐spline add‐on geometries of ggridges v0.5.3 (Wilke, 2021) and ggalt v0.4.0 (Rudis, 2017) respectively. Descriptive statistics were calculated using psych v2.1.9 (Revelle, 2020). 95% confidence intervals were calculated as $\bar{X}$ ± *z* × *SE*, where $\bar{X}$ is the sample mean, *SE* is the standard error of the mean and *z* is the 97.5 percentile point of the standard normal distribution, according to the specific requirements of each model (Bolker, 2021). Plots were aligned and juxtaposed in cowplot v1.1.1 (Wilke, 2020). The base map of Europe in Figure 2a was plotted with rworldmap v1.3‐6 (South, 2011). Illustration and editing were performed in Affinity Designer v1.7.3 (Serif Ltd). The complete data analysis and visualisation pipeline can be downloaded from the open‐access repository at github.com/lukaseamus/CSP.

## 3 RESULTS

### 3.1 Differential carbon export and decomposition speed

Our study site within the latitudinal range overlap (Figure 2a) is currently dominated by the two boreal species *L. digitata* and *L. hyperborea*, with *L. ochroleuca* contributing 22% of forest‐scale kelp density on an annual basis (Table S5). However, at the plant level, *L. ochroleuca* annually exports a similar amount of particulate carbon as *L. hyperborea* and 71% more than *L. digitata* (Tables S5 and S6). Our findings demonstrate that areal particulate carbon export is currently highest for *L. hyperborea* ($\bar{X}$ ± *SE*, 211 ± 27 g C m^−2^ year^−1^, *n* = 108), intermediate for *L. ochroleuca* (127 ± 22 g C m^−2^ year^−1^, *n* = 95) and lowest for *L. digitata* (90 ± 10 g C m^−2^ year^−1^, *n* = 103). Differential plant mass and standing stock further suggest that dislodgement would lead to a similar interspecific contrast in carbon export: highest in *L. hyperborea* (512 ± 20 g plant^−1^, 4.29 ± 0.39 kg m^−2^, *n* = 108), intermediate in *L. ochroleuca* (428 ± 25 g plant^−1^, 1.59 ± 0.23 kg m^−2^, *n* = 96) and lowest in *L. digitata* (315 ± 17 g plant^−1^, 1.42 ± 0.14 kg m^−2^, *n* = 107; Table S6). Importantly, plant density and carbon export also vary seasonally by up to two orders of magnitude (Table S5), with species displaying different patterns throughout the year (Figure S2). Carbon export peaks in spring for *L. hyperborea*, coinciding with the shedding of the old lamina or May cast, followed by two smaller peaks in autumn and winter. The main peak for *L. ochroleuca* is in summer although this species also has secondary and tertiary peaks in autumn and winter. *L. digitata* maintains approximately consistent carbon export throughout the year but reaches a maximum in autumn with very little erosion in winter and spring (Figure S2; Table S5).

**FIGURE 3:**
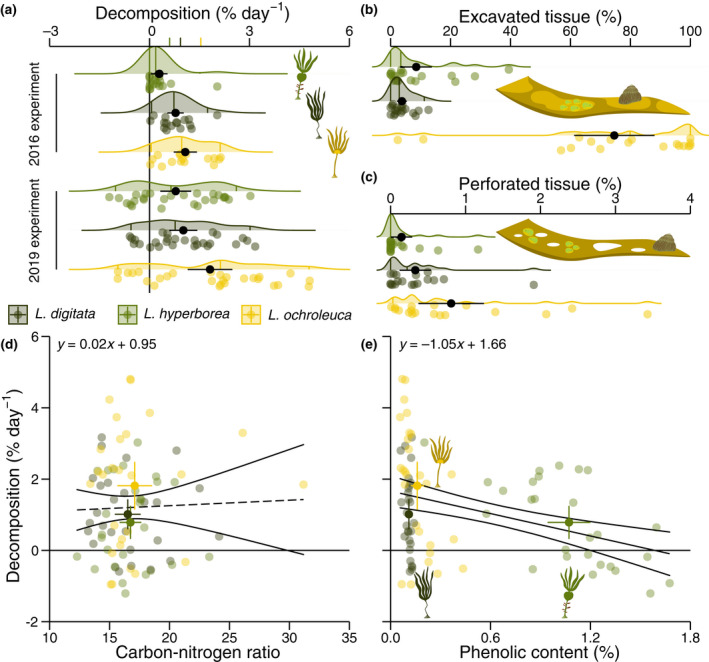
In situ decomposition of Northeast Atlantic *Laminaria* species. (a) Decomposition speed is higher in warm temperate than cold temperate species across experiments. Coloured axis ticks are overall means for each species. (b, c) Differential macrodetritivore grazing activity as evidenced by excavation (b) and perforation (c) scars after 32 days. Raincloud plots show probability density and raw data where vertical bars indicate the median and central 95% of data. Point‐ranges are means and 95% confidence intervals. (d, e) Differential decomposition speed is not explained by elemental stoichiometry (d) and only partially by phenolic concentration (e and Figure S3). Lines show model fits and 95% confidence intervals. Solid lines represent significant slopes at the 95% confidence level, while dashed lines indicate no significant relationship. Point‐ranges are means and 95% confidence intervals.

In addition to the outlined interspecific differences in carbon export, we found that the lability of detritus varies considerably between species. In two independently conducted field experiments on different substrata we found that in situ decomposition occurs 66% and 155% faster in *L. ochroleuca* (1.54 ± 0.23% day^−1^, *n* = 43) than *L. digitata* (0.93 ± 0.14% day^−1^, *n* = 43) and *L. hyperborea* (0.6 ± 0.16% day^−1^, *n* = 43), respectively (Figure 3a; Tables S6 and S7). These data are supported by image analysis, which revealed that *L. ochroleuca* had 7.73–18.77 times larger excavation scars (Figure 3b) and 1.45–4.51 times larger perforation scars (Figure 3c), likely caused by grazing over 1 month on the forest floor (Table S6). Interestingly, detritus of all species displayed the capacity to grow rather than decompose when in the shallow kelp forest (2019 experiment) but not on deeper sediment (2016 experiment; Figure 3a). This may be due to a more suitable photoenvironment in the kelp forest.

Once these differences in decomposition speed were established, we explored whether interspecific biochemical variation was sufficient to explain them. Indeed, we found that detritus of *L. digitata* (27.94 ± 0.39%, *n* = 27) and *L. hyperborea* (28.67 ± 0.41%, *n* = 27) has 27% and 30% higher carbon content than that of *L. ochroleuca* (22.03 ± 1.08%, *n* = 27) suggesting that every one percent increase in carbon content decreases the daily decomposition rate by 0.08% (Table S6). This is supported by somewhat higher lamina carbon content in intact plants of *L. digitata* (28.16 ± 0.56%, *n* = 8) and *L. hyperborea* (30.78 ± 0.92%, *n* = 7) than *L. ochroleuca* (27.38 ± 0.55%, *n* = 6). However, aside from only marginal significance this correlation was not satisfactory due to a similar negative, albeit non‐significant (Table S6), relationship with nitrogen content. Subsequent analysis of the carbon‐nitrogen ratio leaves no doubt that elemental stoichiometry is not sufficiently different between *L. digitata* (16.51 ± 0.55, *n* = 27), *L. hyperborea* (16.74 ± 0.46, *n* = 27) and *L. ochroleuca* (17.09 ± 0.74, *n* = 27) and therefore clearly does not explain interspecific variance in decomposition speed (Figure 3d; Table S6).

*L. hyperborea* (1.07 ± 0.07%, *n* = 27) had 5.69 and 8.75 times higher polyphenolic content than *L. ochroleuca* (0.16 ± 0.02%, *n* = 27) and *L. digitata* (0.11 ± 0.004%, *n* = 27) respectively. While we accordingly found an overall decrease of 1.05% in decomposition speed with every percent increase in soluble phenolic content (Figure 3e; Table S6), *L. digitata* had a lower concentration than expected given its low decomposition rate. Importantly, phenolic content also seems to influence intraspecific variation in decomposition within *L. hyperborea* (–2.44% day^−1^ %^−1^) and *L. ochroleuca* (–9.23% day^−1^ %^−1^; Figure S3; Table S6). Hence, while phenolic concentration is clearly a better predictor of decomposition rate than elmental stoichiometry, it is still not sufficient to explain the majority of variance (Figure 3d–e). In an attempt to more fully explain this remaining variance, we calculated lamina tissue water content as the inverse of dry‐wet mass ratio which we found to be 3% higher in warm temperate kelp (87.32 ± 0.11%, *n* = 94) than *L. digitata* (85.11 ± 0.14%, *n* = 114) and *L. hyperborea* (84.4 ± 0.25%, *n* = 99; Table S6). This may be the most promising predictor of decomposition speed since water content is likely inversely related to tissue toughness. We unfortunately have no direct correlation to support this proposition.

### 3.2 Carbon sequestration potential

**FIGURE 4:**
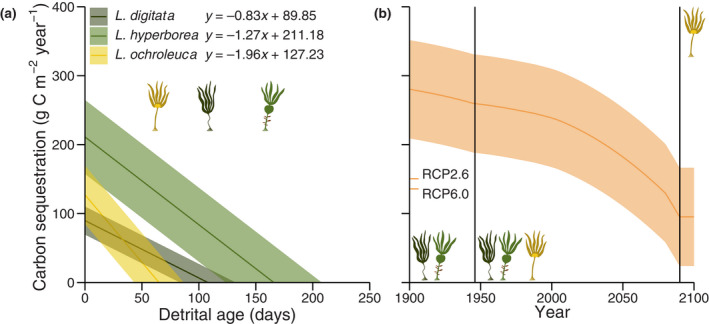
Carbon sequestration potential (CSP) of Northeast Atlantic *Laminaria* species. (a) Decline in presentday CSP with detrital age. Lines and shaded areas are estimates and 95% confidence intervals. Kelp icons indicate when CSP reaches zero. (b) Temporal trend of overall local kelp forest CSP over two centuries according to historical sea surface temperature data and RCP8.5 sea surface temperature predictions for Plymouth Sound (Figure S5). Coloured *y*‐axis ticks indicate end‐of‐century CSP according to alternative scenarios (Figure S6). Since the purpose of these estimates is interspecific comparison, all carbon remaining after 50 days is assumed to count towards CSP because all species still contribute to the detrital carbon pool at that time (a). Lines and shaded areas are estimates and 95% confidence intervals. Kelp icons and vertical lines indicate stages of forest compositional change from before the arrival of *Laminaria ochroleuca* in Plymouth Sound in 1946 to the predicted local extinction of the cold temperate species in 2090 (cf. Figure 2). RCP, representative concentration pathway.

On the basis of the presented empirical evidence, we suggest that local compositional change of Northeast Atlantic *Laminaria* forests via climate‐driven poleward range shifts is a mechanism that has the potential to reduce regional CSP. We estimated linear relationships between current species‐specific CSP (g C m^−2^ year^−1^) and detrital age (methods Section 2.3.1). As expected, the CSP of *L. ochroleuca* declines 54%–135% faster per day than that of the cold temperate species (Figure 4a). Consequently, *L. digitata* and *L. hyperborea* CSP reach zero after 108 ± 12 days and 166 ± 21 days, on average 43 days and 101 days later than that of their warm temperate congener (65 ± 11 days; Figure 4a). Because decomposition is logically assumed to be proportional to the magnitude of the detrital pool, this contrast is maintained throughout the year (Figure S4a) despite strong seasonal variation in carbon export (Figure S2).

Climate change is shifting the outlined species distributions (Figure 2a), based on differential temperature tolerance (Figure 2b), which is leading to a restructuring of Northeast Atlantic kelp forest composition. Therefore we modelled the effects of this climate‐driven ecosystem alteration on forest carbon export and CSP through time, on the basis of species‐specific temperature tolerances, historical temperature data and RCP temperature predictions for the region (methods Section 2.3.2, Figure S5). The purpose of this thought experiment is to highlight interspecific diversity and it should thus be inclusive of all species. We therefore assume that all carbon remaining after 50 days, when no species' exported carbon has fully decomposed (Figure 4a), constitutes CSP. Our estimates suggest that despite fluctuating but stable carbon export across both centuries (Figure S6), forest CSP declined by around 0.17% per year in the past and will likely continue to do so under RCP8.5 at a rate that is 3.04 times higher (0.69% year^−1^, Figure 4b). RCP6.0 and RCP2.6 are predicted to reduce present CSP 31% and 43% less by the end of the century (Figure 4b; Figure S7), which suggests that climate change mitigation may alleviate forest CSP loss. Hence, we predict that through a vicious circle, climate‐driven range shifts may lead to a local reduction of a climate change mitigating ecosystem service. Of course it is likely that it takes longer than 50 days for kelp detritus to reach sedimentary carbon sinks and hence only boreal species would contribute to carbon sequestration (Figure 4a); however, this would only exacerbate the predicted trend.

### 3.3 Detrital carbon assimilation and potential feedback loops

**FIGURE 5:**
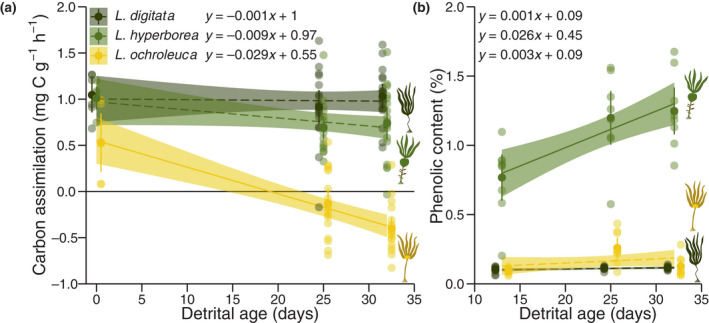
Consequences of decomposition for Northeast Atlantic *Laminaria* species detritus. (a) Decomposition has a contrasting effect on the net primary production of cold temperate and warm temperate species at 50.4 μmol photons m^−2^ s^−1^. Point‐ranges indicate means and 95% confidence intervals. Lines and shaded areas are model predictions and 95% confidence intervals. Solid lines represent significant slopes at the 95% confidence level, while dashed lines indicate no significant change over time. Carbon assimilation was calculated from oxygen production, assuming a photosynthetic quotient of 1, and is given per g of dry mass. (b) Decomposition only increases the proportional chemical defences of *L. hyperborea*. Point‐ranges indicate means and 95% confidence intervals. Lines and shaded areas are model predictions and 95% confidence intervals. Solid lines represent significant slopes at the 95% confidence level, while dashed lines indicate no significant change over time.

We present the first interspecific comparsion of detrital photophysiological viability in situ and found that *L. digitata* and *L. hyperborea* maintained NPP at 0.98 ± 0.05 and 0.76 ± 0.05 mg C g^−1^ h^−1^ (*n* = 42) over 1 month on the forest floor (Figure 5a; Table S6). In contrast, photosynthesis of *L. ochroleuca*, which was initially 48%–50% lower than that of its cold temperate congeners (0.53 ± 0.16, *n* = 6), declined at a rate of 0.03 mg C g^−1^ h^−1^ per day (Figure 5a; Table S6). Consequently, *L. ochroleuca* detritus is predicted to emit carbon after 20 days and assimilates 1.43 ± 0.09 and 1.14 ± 0.11 mg C g^−1^ h^−1^ (*n* = 18) less than *L. digitata* and *L. hyperborea* after 1 month, respectively. GPP displayed a similar pattern (Figure S8; Table S6), indicating that increased microbial respiration is not the primary driver of the observed pattern in NPP and the photosynthetic apparatus of *L. ochroleuca* is in fact predicted to fail completely after 36 days of decomposition.

These results have major implications for detrital carbon assimilation in different photoenvironments, which in turn affects decomposition. Using local photon flux density data and photosynthesis‐irradiance relationships (method Section 2.3.3), we estimate that cold and warm temperate species can increase their respective yearly carbon export by 53%–87% and 4% via detrital carbon assimilation. On an annual basis, the boreal kelps studied can potentially assimilate 5%–199% more carbon during their detrital phase than *L. ochroleuca*. Interestingly, cold temperate species have higher cumulative carbon assimilation throughout the year except for summer, when *L. ochroleuca* detritus could potentially assimilate 1.95–4.52 times more carbon on its way to local sedimentary carbon sinks (Figure S4b). Across species, carbon assimilation generally declines from spring through to winter rather than peaking in summer as expected from seasonal light availability (Figure S4b). Depending on the light regime, disintegration of the photosynthetic apparatus of *L. ochroleuca* due to decomposition is a significant positive feedback loop that may further exacerbate interspecific differences in CSP beyond those reported above (Figure 4).

We also uncovered a potential negative feedback loop linking detrital residence time to decomposition. Proportional chemical defence compounds (polyphenols) increase at a rate of 0.03% day^−1^ in *L. hyperborea* while they remained low and unchanged in the other species (Figure 5b; Table S6). Since phenolic content may decrease decomposition (Figure 3e), an induced response to detritivore activity provides a tantalising explanation for this observation. This would mean that rather than becoming accessible to more consumers through decomposition, detritus of *L. hyperborea* either defends itself and becomes less palatable, or becomes less palatable as polyphenolics are lost less quickly than other macrolecules, therefore maintaining integrity for longer. Carbon content increases at a rate of 0.14% day^−1^ in *L. digitata* and remains unchanged in the other *Laminaria* species (Table S6) but generally tends to diverge between cold and warm temperate species with detrital age. Since nitrogen content behaves similarly, the carbon–nitrogen ratio declines at an exponential rate of 0.005 day^−1^ in all species (Table S6).

## 4 DISCUSSION

Analogous to previous studies on seagrasses (Cebrián et al., 1997; Lavery et al., 2013) and mangroves (Atwood et al., 2017; Li et al., 2018), we report strong interspecific differences in local macroalgal CSP using a kelp forest of the genus *Laminaria* as a model system. To our knowledge, this is also the first detailed documentation of carbon flux (primary production, standing stock, particulate carbon export and remineralization) through a single kelp forest. We show that in the southern UK, warm temperate kelp decomposes faster than cold temperate species (Figure 3) and therefore has a lower CSP (Figure 4a). This difference is likely upheld by a variety of mechanistic drivers including variance in phenolic concentration (Figures 3e and 5b), tissue water content and detrital photosynthesis (Figure 5a). Our data suggest that local to regional kelp forest CSP may already have declined and probably will continue to do so with further poleward movement of *Laminaria* species under continued ocean warming. These potential repercussions for ecosystem functioning represent just one of many vicious circles, since climate change is already reducing the extent of many temperate kelp forests (Smale, 2020) and their CSP is additionally expected to be diminished by climate‐driven phase shifts (Krumhansl et al., 2014; Pessarrodona et al., 2021), heatwaves (Smale et al., 2019), forest miniaturisation (King et al., 2020; Pessarrodona et al., 2018) and coastal darkening (Blain et al., 2021).

Our modelled interspecific contrasts in CSP are likely underestimates since a range of factors may further diminish the CSP of warm temperate kelp. First, in stark contrast to *L. hyperborea* (Smale et al., 2015; Teagle & Smale, 2018) and *L. digitata* (Figure S9), *L. ochroleuca* is depauperate of epiphytes (mostly *Palmaria palmata* in terms of biomass), which reduces the total carbon stock and hence CSP of forests formed by this species (Smale et al., 2015; Teagle & Smale, 2018). Second, *L. hyperborea* is unique in shedding over 60% of its laminar biomass, termed the May cast, in spring (Figure S2; Lüning, 1969; Pedersen et al., 2020), which has been suggested to temporarily overwhelm consumers, thus increasing CSP (Pedersen et al., 2020). Third, sea surface temperature is approximately 7°C warmer during peak carbon export by *L. ochroleuca* than *L. hyperborea* (Figures S2 and S4), which leads to elevated bacterial activity (White et al., 1991), alginate degrading enzymes (Minich et al., 2018) and amphipod grazing (Gilson et al., 2021) and a subsequent increase in decomposition rate. Fourth, *L. hyperborea* detritus remains intact for at least 6 months on shallow sediment due to exponential decay (de Bettignies, Dauby, Thomas, et al., 2020), so our linear model may overestimate this species' long‐term decomposition speed. Finally, greater longevity of cold temperate kelp detritus likely allows for export to hypoxic regions of the deep sea where decomposition is slower (Pedersen et al., 2021). In contrast, the detrial carbon pool of *L. ochroleuca* is predicted to be remineralized within 65 days, precluding long‐distance transport.

The extent of the implications of such changes on a global scale remains doubtful, particularly due to uncertainty surrounding future kelp distribution in the Arctic under different climate change scenarios. If the total area of kelps across the Arctic maximally increases by 118,500 km^2^ (Assis et al., 2022), hempisphere‐scale kelp CSP might actually stay stable or increase. This scenario is imaginable since *L. hyperborea* and *L. digitata* and their arctic congener *Laminaria solidungula*, the CSP of which is unknown, could expand their ranges poleward, potentially mitigating or overcompensating the loss of trailing edge populations. If, however, there is little to no novel arctic habitat for boreal and arctic kelps to expand into (Bringloe et al., 2022), then their global range will contract and their potential contribution to carbon sequestration diminish, mirroring the local processes we predict. This scenario is arguably more likely due to decreased salinity and increased turbidity through glacial and permafrost melt in combination with coastal erosion, and photoperiod‐mediated disruption of growth and reproductive cycles in taxa adapted to lower latitudes (Bringloe et al., 2022; Martins et al., 2022). We nevertheless emphasise that our predictions of declining CSP beyond the local scale represent a thought experiment, intended to stimulate further study and debate rather than provide firm predictions, being based as they are on finite empirical data. The outlined contradictory species distribution model predictions clearly point towards the scarcity of empirical data on the distribution and ecophysiology of arctic kelps and quantifcation of their CSP emerges as a research priority.

Our empirical findings on carbon remineralization are supported by the feeding preference of the key kelp detritivores (de Bettignies, Dauby, Lepoint, et al., 2020) *Steromphala umbilicalis* (Gilson et al., 2021), *S. cineraria*, and *P. pellucida* (Hargrave et al., 2017; Pessarrodona et al., 2019; Smale et al., 2015) for *L. ochroleuca* compared to boreal species. However, our results contradict the paradigm that elemental stoichiometry is the principal driver of plant decomposition (Enríquez et al., 1993). This may be explained by the low representation of macroalgal data in meta‐analyses. Alternatively, elemental variance within *Laminaria* may not be large enough to affect detritivores and saprotrophs. The low polyphenol levels reported for *L. digitata* are comparable to previous data (Hereward et al., 2018), which leaves the enigma of why this species has similarly slow decomposition to *L. hyperborea*. Its lower lamina tissue water content may supply part of the answer, but it is probable that other structural differences and/or biochemical defences are involved. For instance, *L. digitata* utilises reactive oxygen species and iodine in defence (Cosse et al., 2009), which is likely relevant since iodine plays a crucial role in structuring the saprotrophic community in the early stages of decomposition (de Bettignies, Dauby, Thomas, et al., 2020). Another probable mechanistic driver is the slower annual growth rate of boreal kelps (Pessarrodona et al., 2019), which negatively correlates with decomposition rate across plant groups (Cebrián & Duarte, 1995).

Our data on the effect of decomposition on detrital photosynthesis and phenolic content, albeit somewhat scarce, are supported by several lines of evidence. Warm temperate kelp has a 42%–45% lower total photosynthetic pigment concentration than cold temperate kelps (Wright & Foggo, 2021), suggesting it has lower photosynthetic capacity which is in line with the 48%–50% lower photosynthetic activity reported here. Although Frontier, de Bettignies, et al. (2021) found no difference in net and gross primary production and photosynthetic efficiency (chlorophyll fluorescence) between in vitro *L. hyperborea* and *L. ochroleuca*, a subsequent field experiment by the same authors suggests declining photosynthetic efficiency with detrital age in *L. ochroleuca* and no change in *L. hyperborea* (Frontier, Mulas, et al., 2021). This case supports our findings and highlights that in situ experimental validation is essential. Over the first 6 weeks of decomposition, phenolic content was previously found to increase with detrital age in *L. hyperborea* at a similar rate (0.06% day^−1^) to that reported here (0.03% day^−1^; de Bettignies, Dauby, Thomas, et al., 2020). Furthermore, wounding is known to cause the production of phlorotannins in this species (Halm et al., 2011).

Despite our prediction of diminishing local CSP in a warmer climate, there is scope for the preservation of this regulating ecosystem service. Besides the need for reducing anthropogenic carbon emission emphasised here, kelp blue carbon function could potentially be maintained through marine protected areas (Ling et al., 2009), reforestation (Layton et al., 2020) and hybridisation (Martins et al., 2019) alongside restrictions on eutrophication and sedimentation (Blain et al., 2021; Pessarrodona et al., 2021). The latter have the potential to be innovative alternatives for Annex I parties like the United Kingdom, United States, and Australia, which have few ocean‐based NDCs (Gallo et al., 2017). Enhancement of macroalgal CDR, termed seaweed offsetting (Froehlich et al., 2019) or ocean afforestation (Bach et al., 2021), is currently heatedly debated on a weak empirical foundation. Both proponents (Froehlich et al., 2019) and opponents (Bach et al., 2021) have made substantive claims on the basis of extrapolation from single, fundamentally different macroalgal genera (*Macrocystis* vs. *Sargassum*). Here we show that in fact interspecific diversity in CSP is substantial within a single macroalgal genus, rendering this debate inutile without further empirical evidence.

**FIGURE 6:**
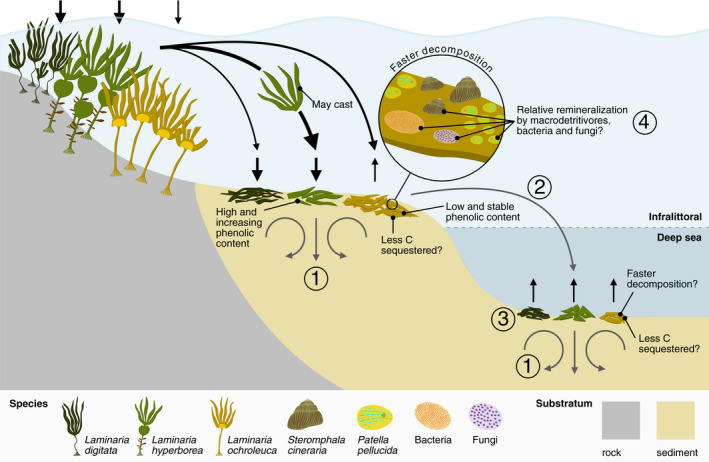
Key findings and remaining knowledge gaps. Black arrows indicate particulate carbon fluxes that were measured in this study, while grey arrows indicate those that have yet to be assessed. Knowledge gaps are, therefore, ① carbon sequestration in shallow and deep sediments, ② carbon export to and ③ decomposition in the deep ocean and ④ carbon remineralization by microbial saprotrophs and macrodetritivores.

Our results clearly show that developing accurate estimates of macroalgal contribution to global carbon sequestration will need a more nuanced understanding of different factors causing variability. Therefore, important future research goals should include (1) quantifying the effect of forest state and composition on regional carbon burial, (2) measuring variance in decomposition speed between additional species and across depths/photoenvironments, (3) determining the CSP of arctic kelps (e.g. *L. solidungula* in our model system), and (4) unravelling microbial detrital pathways in the shallow and deep ocean in addition to addressing various knowledge gaps that are specific to our model system (Figure 6). To achieve these goals and understand how climate change is affecting marine ecosystem services more broadly, we argue that interspecific diversity should always be considered and more multidisciplinary research is required to bridge the void between disconnected scientific branches. Specifically, interdisciplinary links between physiology, ecology, biogeography, biogeochemistry, physics, and genetics are now needed more than ever to understand the function of marine plants in a rapidly changing environment.

## AUTHOR CONTRIBUTIONS

Luka Seamus Wright and Andy Foggo designed the study and collected data on decomposition, phenolic content, elemental stoichiometry and photosynthesis in 2019. Albert Pessarrodona collected data on decomposition, standing stock and carbon export in 2016. Luka Seamus Wright analysed and visualised all data and performed all modelling and estimation. Luka Seamus Wright wrote the draft version of the manuscript. All authors edited and approved the final manuscript.

## CONFLICT OF INTEREST

The authors declare no competing interests.

## Supporting information


Appendix S1.

## Data Availability

All data sets and annotated R scripts written for this study are available in the open‐access repository at github.com/lukaseamus/CSP. Data can also be downloaded at https://doi.org/10.5061/dryad.m905qfv40. We place no restrictions on data and code availability. Luka Seamus Wright maintains these repositories and may be contacted for queries or further requests.
