# Supplementary material for: Climate‐driven shifts in kelp forest composition reduce carbon sequestration potential
Source: Glob Chang Biol. 2022 Jul 5;28(18):5514–31. doi: 10.1111/gcb.16299 (PMC9545355; doi:10.1111/gcb.16299)
Supplement: Supplementary file 1 — Appendix S1. [file GCB-28-5514-s001.pdf]

Climate-driven shifts in kelp forest composition reduce carbon sequestration potential

Luka Seamus Wright[1,2] * (0000-0002-1273-6256), Albert Pessarrodona[2] (0000-0002-6057-9937) and Andy Foggo[1] (0000-0002-0280-0824)

[1]Marine Biology and Ecology Research Centre, University of Plymouth, Plymouth, PL4 8AA, United Kingdom.
[2]Oceans Institute, University of Western Australia, Perth, WA 6009, Australia.
*corresponding author: luka@wright.it, luka.wright@research.uwa.edu.au

Supplementary information
1. Tables

Table S1. Estimated global marine net primary production (NPP) and carbon sequestration potential (CSP) of major marine autotrophic groups. Confidence intervals are 95% confidence intervals, calculated as mean ± $z \times$ standard error, where z is the 97.5 percentile point of the standard normal distribution. In the case of seagrasses, CI is half range uncertainty because standard errors were not reported. % indicates the percentage of NPP that is sequestered.

	NPP (Gt C yr^{-1})		CSP (Gt C yr^{-1})		
**Plant**	**Mean**	**Confidence interval**	**Mean**	**Confidence interval**	**CSP (%)**
Seagrasses	0.315[1]	0.14–0.49[1]	0.067[1, 2]	0.03–0.104[1, 2]	21.2
Macroalgae	1.521[3]	1.476–1.566[3]	0.173[3]	0.163–0.183[3]	11.4
Phytoplankton	58[4]	51–65[4]	0.232[4–6]	0.204–0.26[4–6]	0.4

Table S2. Macroalgal species feeding into parameters of the most recent estimates of global macroalgal carbon sequestration potential[3]. Studies of whole communities with no mention of species or genera are not included below. Species marked in bold were studied as distinct species rather than as part of bulk community measurements. When a meta-analysis was cited by Krause-Jensen and Duarte[3], both the meta-analysis and the original study are referenced. All taxonomic names were updated to current standards using WoRMS[7].

**Parameter**	**Species (order, class)**
Carbon assimilation	***Callithamnion corymbosum* (Ceramiales, Florideophyceae)[8, 9]**
	***Ceramium diaphanum* (Ceramiales, Florideophyceae)[8, 9]**
	***Ceramium virgatum* (Ceramiales, Florideophyceae)[8, 9]**
	***Cladophora* sp. (Cladophorales, Ulvophyceae)[8, 9]**
	***Cladostephus spongiosus f. verticillatus* (Sphacelariales, Phaeophyceae)[8, 9]**
	***Dictyota fasciola* (Dictyotales, Phaeophyceae)[8, 9]**
	***Fucus* sp. (Fucales, Phaeophyceae)[10, 11]**
	***Fucus vesiculosus* (Fucales, Phaeophyceae)[10, 12]**
	***Halimeda opuntia* (Bryopsidales, Ulvophyceae)[10, 13]**
	***Hydrolithon boergesenii* (Corallinales, Florideophyceae)[10, 14–16]**
	***Hydrolithon gardineri* (Corallinales, Florideophyceae)[10, 15, 16]**
	***Laminaria digitata* (Laminariales, Phaeophyceae)[10, 17, 18]**
	***Laminaria pallida* (Laminariales, Phaeophyceae)[10, 19]**
	***Laurencia obtusa* (Ceramiales, Florideophyceae)[8, 9]**
	***Lithophyllum intermedium* (Corallinales, Florideophyceae)[10, 20]**
	***Lithophyllum* spp. (Corallinales, Florideophyceae)[10, 20]**
	***Macrocystis pyrifera* (Laminariales, Phaeophyceae)[10, 12, 21]**
	***Neogoniolithon fosliei* (Corallinales, Florideophyceae)[10, 20]**
	***Porolithon antillarum* (Corallinales, Florideophyceae)[10, 20]**
	***Porolithon onkodes* (Corallinales, Florideophyceae)[10, 15, 16, 22]**
	Porolithon spp. (Corallinales, Florideophyceae)[10, 23]
	***Saccharina latissima* (Laminariales, Phaeophyceae)[10, 24]**
	***Saccharina longicruris* (Laminariales, Phaeophyceae)[10, 17, 18, 25]**
	***Sargassum platycarpum* (Fucales, Phaeophyceae)[10, 26]**
	***Sporolithon* erythraeum (Sporolithales, Florideophyceae)[10, 15, 16]**
	***Sporolithon ptychoides* (Sporolithales, Florideophyceae)[10, 14]**
	***Sporolithon* sp. (Sporolithales, Florideophyceae)[10, 27]**
	***Treptacantha barabata* (Fucales, Phaeophyceae)[8, 9]**

	***Ulva* sp. (Ulvales, Ulvophyceae)**[8, 9]
	***Vertebrata subulifera* (Ceramiales, Florideophyceae)**[8, 9]
Total carbon export	***Acanthophora spicifera* (Ceramiales, Florideophyceae)**[28, 29]
	***Amphiroa fragilissima* (Corallinales, Florideophyceae)**[28, 29]
	***Canistrocarpus cervicornis* (Dictyotales, Phaeophyceae)**[28, 29]
	Caulerpa cupressoides (Bryopsidales, Ulvophyceae)[28, 30]
	Caulerpa racemosa (Bryopsidales, Ulvophyceae)[28, 30]
	***Centroceras clavulatum* (Ceramiales, Florideophyceae)**[28, 29]
	Centroceras clavulatum (Ceramiales, Florideophyceae)[28, 30]
	***Cladophora albida* (Cladophorales, Ulvophyceae)**[28, 31]
	Codium tomentosum (Bryopsidales, Ulvophyceae)[28, 30]
	Dermonema virens (Nemaliales, Florideophyceae)[28, 30]
	Dictyopteris delicatula (Dictyotales, Phaeophyceae)[28, 32]
	Dictyota bartayresiana (Dictyotales, Phaeophyceae)[28, 32]
	Dictyota dichotoma (Dictyotales, Phaeophyceae)[28, 32]
	Dictyota guineënsis (Dictyotales, Phaeophyceae)[28, 32]
	Dictyota implexa (Dictyotales, Phaeophyceae)[28, 32]
	Dictyota jamaicensis (Dictyotales, Phaeophyceae)[28, 32]
	Dictyota mertensii (Dictyotales, Phaeophyceae)[28, 32]
	Dictyota pinnatifida (Dictyotales, Phaeophyceae)[28, 32]
	***Ecklonia maxima* (Laminariales, Phaeophyceae)**[28, 33]
	***Gelidiella acerosa* (Gelidiales, Florideophyceae)**[28, 29]
	Gracilaria corticata (Gracilariales, Florideophyceae)[28, 30]
	Gracilaria foliifera (Gracilariales, Florideophyceae)[28, 30]
	Gracilaria textorii (Gracilariales, Florideophyceae)[28, 30]
	Gracilariopsis lemaneiformis (Gracilariales, Florideophyceae)[28, 30]
	Gracilariopsis longissima (Gracilariales, Florideophyceae)[28, 30]
	Halimeda tuna (Bryopsidales, Ulvophyceae)[28, 30]
	***Hypnea musciformis* (Gigartinales, Florideophyceae)**[28, 29]
	***Hypnea spinella* (Gigartinales, Florideophyceae)**[28, 29]
	Laurencia obtuse (Ceramiales, Florideophyceae)[28, 30]
	Laurencia sp. (Ceramiales, Florideophyceae)[28, 30]
	***Macrocystis pyrifera* (Laminariales, Phaeophyceae)**[28, 34]
	Melanothamnus somalensis (Ceramiales, Florideophyceae)[28, 30]
	Padina gymnospora (Dictyotales, Phaeophyceae)[28, 30]
	Padina sanctae-crucis (Dictyotales, Phaeophyceae)[28, 32]
	Padina sp. (Dictyotales, Phaeophyceae)[28, 30]
	Padina tetrastromatica (Dictyotales, Phaeophyceae)[28, 30]
	***Palisada perforata* (Ceramiales, Florideophyceae)**[28–30]
	Rhodymenia sp. (Rhodymeniales, Florideophyceae)[28, 30]
	***Saccharina longicruris* (Laminariales, Phaeophyceae)**[17, 28]
	Sarconema filiforme (Gigartinales, Florideophyceae)[28, 30]
	Stoechospermum polypodioides (Dictyotales, Phaeophyceae)[28, 30]
	Stypopodium zonale (Dictyotales, Phaeophyceae)[28, 32]
	Ulva flexuosa (Ulvales, Ulvophyceae)[28, 30]
	Ulva intestinalis (Ulvales, Ulvophyceae)[28, 30]
	Ulva lactuca (Ulvales, Ulvophyceae)[28, 30]
	Ulva linza (Ulvales, Ulvophyceae)[28, 30]
Dissolved carbon export	***Caulerpa prolifera* (Bryopsidales, Ulvophyceae)**[35]
	***Caulerpa racemosa* (Bryopsidales, Ulvophyceae)**[35]
	***Fucus serratus* (Fucales, Phaeophyceae)**[35]
	***Fucus vesiculosus* (Fucales, Phaeophyceae)**[35]
	***Halimeda tuna* (Bryopsidales, Ulvophyceae)**[35]
	***Laminaria* sp. (Laminariales, Phaeophyceae)**[35]
Deep sea carbon export	*Dictyopteris delicatula* (Dictyotales, Phaeophyceae)[32]
	Dictyota bartayresiana (Dictyotales, Phaeophyceae)[32]
	Dictyota dichotoma (Dictyotales, Phaeophyceae)[32]
	Dictyota guineënsis (Dictyotales, Phaeophyceae)[32]
	Dictyota implexa (Dictyotales, Phaeophyceae)[32]
	Dictyota jamaicensis (Dictyotales, Phaeophyceae)[32]
	Dictyota mertensii (Dictyotales, Phaeophyceae)[32]
	Dictyota pinnatifida (Dictyotales, Phaeophyceae)[32]
	Padina sanctae-crucis (Dictyotales, Phaeophyceae)[32]
	***Sargassum* spp. (Fucales, Phaeophyceae)**[36]

	Stypopodium zonale (Dictyotales, Phaeophyceae)[32]
Carbon burial	***Gracilaria* spp. (Gracilariales, Florideophyceae)**[37]

Table S3. Scientific literature and records on the past, present and future biogeographic distribution of Northeast Atlantic *Laminaria* species and the northern biogeographic boundary of warm temperate kelps. The present study concerns compositional change in a mixed kelp forest at the range overlap of all species. Therefore, the focus of the presented past and future species distribution data lies in the trailing range edges of *L. digitata* and *L. hyperborea* and the leading range edge of *L. ochroleuca*. Nevertheless, it should be remarked that historical records[38] and recent evidence[39] point towards a similar shift of the trailing edge of *L. ochroleuca* and the leading edges of *L. digitata*[40] and *L. hyperborea*[41] are also likely shifting north.

Species	Present	Past	Future	Boundary
L. digitata	OBIS[42], GBIF[43], NBN[44], OSPAR (unpublished data)	van den Hoek and Donze[45], Lüning[46]	Raybaud et al.[47], Assis et al.[48]	
L. hyperborea	OBIS[49], GBIF[50], NBN[51], Assis et al.[52], Casado-Amezúa et al.[53], OSPAR (unpublished data)	GBIF[54], Lüning[46]	Assis et al.[48], Assis et al.[55]	
L. ochroleuca	Casado-Amezúa et al.[53], OBIS[56], GBIF[57], NBN[58], Schoenrock et al.[59], Voerman et al.[60], Giaccone[61], OSPAR (unpublished data)	Schoenrock et al.[59], Parke[62]	Assis et al.[48], Franco et al.[63]	Lüning[46], Forbes[64]

Table S4. Temperature tolerance of Northeast Atlantic *Laminaria* species. Temperature tolerance refers to the ability to grow for sporophytes and maintenance of fertility for gametophytes. The present study concerns changes in kelp forest composition due to differential thermal tolerance of component species. Therefore, the data presented here are those most relevant to environmental change. Nevertheless, it should be remarked that *Laminaria* species gametophytes can survive a greater temperature range than sporophytes[65] and the fertility of sporophytes is sometimes more temperature-limited than that of gametophytes[66].

Species	Stage	Optimum (°C)	Highest tolerance (°C)	Lowest tolerance (°C)
L. digitata	Sporophyte	10[67–69]	20[67–70]	–2[70]
	Gametophyte	10[69–72]	17[69, 72, 73]	0[74]
L. hyperborea	Sporophyte	15[67–69]	20[67–69]	–1.5[75]
	Gametophyte	15[69, 71]	18[69, 73]	2[69, 70]
L. ochroleuca	Sporophyte	15[69, 76, 77]	25[63]	5[69, 76]
	Gametophyte	15[77, 78]	21[69, 77]	10[70, 77, 78]

Table S5. Seasonal and annual sporophyte densities and particulate organic carbon (POC) export of Northeast Atlantic *Laminaria* species, given as means ± standard errors, in the kelp forest at West Hoe between January 2016 and March 2017. Sporophyte densities were adjusted for vertical kelp forest zonation and are given as forest-wide densities. These empirical data were used to derive an estimate of areal carbon export. Note that the given overall sample sizes for sporophyte densities and carbon export do not correspond directly to the means and standard errors since they were summed across months and derived via the variance sum law respectively as described in the methods.

Species	Season	n	Density (plants m^{-2})	Forest density (plants m^{-2})	n	POC export (g C $plant^{-1}$)	POC export (g C m^{-2})
L. digitata	Annual	75	22.51 ± 1.85	4.5 ± 0.37	103	19.96 ± 1.62	89.85 ± 10.4
	Spring	20	28.8 ± 4.27	5.76 ± 0.85	24	4.28 ± 1.17	24.63 ± 7.74
	Summer	27	12 ± 1.91	2.4 ± 0.38	28	7.13 ± 0.82	17.11 ± 3.38
	Autumn	15	32.8 ± 2.54	6.56 ± 0.51	22	7.28 ± 0.73	47.74 ± 6.05
	Winter	13	22.77 ± 4.19	4.55 ± 0.84	29	1.28 ± 0.24	5.81 ± 1.53
L. hyperborea	Annual	73	10.47 ± 0.87	8.37 ± 0.69	108	25.22 ± 2.5	211.18 ± 27.37
	Spring	20	11.6 ± 1.59	9.28 ± 1.27	30	13.99 ± 1.78	129.84 ± 24.39
	Summer	26	7.38 ± 1.5	5.91 ± 1.2	26	1.27 ± 0.39	7.49 ± 2.81
	Autumn	13	15.08 ± 1.98	12.06 ± 1.59	25	7.13 ± 1.56	85.98 ± 22.08
	Winter	14	10.29 ± 1.44	8.23 ± 1.15	27	2.83 ± 0.71	23.33 ± 6.73
L. ochroleuca	Annual	73	4.66 ± 0.6	3.73 ± 0.48	95	34.15 ± 3.75	127.23 ± 21.59
	Spring	20	3.2 ± 0.9	2.56 ± 0.72	26	3.56 ± 0.53	9.11 ± 2.93

Summer	26	5.38 ± 1.09	4.31 ± 0.87	19	17.76 ± 3.49	76.49 ± 21.74
Autumn	13	6.46 ± 1.9	5.17 ± 1.52	24	10.33 ± 1.19	53.42 ± 16.95
Winter	14	3.71 ± 0.89	2.97 ± 0.71	26	2.49 ± 0.42	7.41 ± 2.18

Table S6. Linear model (LM), generalised linear model (GLM), generalised least squares (GLS) and linear mixed effects (LME) results obtained from omnibus and pairwise tests. The latter are only given when more than two categories need comparing. When the interaction term was found to be significant, it is reported and statistics for the slope (i.e. the continuous explanatory variable) are given for each species. Details on the construction of each model can be found in the R code (github.com/lukaseamus/CSP).

Variables	Model	Test	n	df	*F*	X^2	*t*	p	
**Carbon export (g C plant^{-1} d^{-1})**									
Species	GLM	Omni	306	2		9.99		0.007	**
L. digitata < *L. hyperborea*		Pairs					2.08	0.04	*
L. digitata < *L. ochroleuca*							3.16	0.002	**
L. hyperborea = *L. ochroleuca*							1.15	0.25	
**Plant mass (g)**									
Species	LM	Omni	311	2	23.57			< 0.001	***
L. digitata < *L. hyperborea*		Pairs					6.85	< 0.001	***
L. digitata < *L. ochroleuca*							3.79	< 0.001	***
L. hyperborea > *L. ochroleuca*							2.86	0.005	**
**Decomposition (% d^{-1})**									
Species	LM	Omni	129	2	7.13			0.001	**
Experiment				1	5.28			0.02	*
Residuals				125					
L. digitata = *L. hyperborea*		Pairs					1.29	0.2	
L. digitata < *L. ochroleuca*							2.43	0.02	*
L. hyperborea < *L. ochroleuca*							3.72	< 0.001	***
**Decomposition (% d^{-1})**									
Species	GLS	Omni	81	2		0.02		0.99	
Phenolic content × Species				2		8.16		0.02	*
Phenolic content (*L. digitata*)				1		3.47		0.06	
Phenolic content (*L. hyperborea*)				1		21.02		< 0.001	***
Phenolic content (*L. ochroleuca*)				1		10.55		0.001	**
**Decomposition (% d^{-1})**									
Phenolic content	GLS	Omni	81	1		15.63		< 0.001	***
**Decomposition (% d^{-1})**									
Carbon content	LM	Omni	81	1	5.75			0.02	*
**Decomposition (% d^{-1})**									
Nitrogen content	LM	Omni	81	1	3.69			0.06	
**Decomposition (% d^{-1})**									
Carbon-nitrogen ratio	LM	Omni	81	1	0.08			0.77	
**Excavated tissue (%)**									
Species	GLM	Omni	54	2		104.94		< 0.001	***
L. digitata < *L. hyperborea*		Pairs					2.48	0.02	*
L. digitata < *L. ochroleuca*							9.87	< 0.001	***
L. hyperborea < *L. ochroleuca*							7.39	< 0.001	***
**Perforated tissue (%)**									
Species	GLM	Omni	54	2		12.9		0.002	**
L. digitata = *L. hyperborea*		Pairs					1.13	0.26	
L. digitata < *L. ochroleuca*							2.35	0.02	*
L. hyperborea < *L. ochroleuca*							3.49	0.001	**
**Tissue water content (%)**									
Species	GLS	Omni	307	2		210.03		< 0.001	***
L. digitata > *L. hyperborea*		Pairs					2.46	0.01	*
L. digitata < *L. ochroleuca*							12.2	< 0.001	***
L. hyperborea < *L. ochroleuca*							10.6	< 0.001	***
**Net carbon assimilation (mg C g^{-1} h^{-1})**									
Species	LM	Omni	126	2	3.95			0.02	*
Detrital age × Species				2	9.27			< 0.001	***
Detrital age (*L. digitata*)				1	0.02			0.88	
Detrital age (*L. hyperborea*)				1	3.36			0.07	

Detrital age (*L. ochroleuca*)				1	36.77			< 0.001	***
Residuals				120					
L. digitata = *L. hyperborea*		Pairs					0.17	0.86	
L. digitata > *L. ochroleuca*							2.52	0.01	*
L. hyperborea > *L. ochroleuca*							2.34	0.02	*
**Respiration (mg C g^{-1} h^{-1})**									
Species	GLS	Omni	126	2		42.68		< 0.001	***
Detrital age × Species				2		25.53		< 0.001	***
Detrital age (*L. digitata*)				1		21.69		< 0.001	***
Detrital age (*L. hyperborea*)				1		7.32		0.007	**
Detrital age (*L. ochroleuca*)				1		1.59		0.21	
L. digitata < *L. hyperborea*		Pairs					6.06	< 0.001	***
L. digitata < *L. ochroleuca*							3.39	0.001	**
L. hyperborea = *L. ochroleuca*							0.57	0.57	
**Gross carbon assimilation (mg C g^{-1} h^{-1})**									
Species	LM	Omni	126	2	3.72			0.03	*
Detrital age × Species				2	13.62			< 0.001	***
Detrital age (*L. digitata*)				1	1.45			0.23	
Detrital age (*L. hyperborea*)				1	8.54			0.004	**
Detrital age (*L. ochroleuca*)				1	37.94			< 0.001	***
Residuals				120					
L. digitata < *L. hyperborea*		Pairs					2.36	0.02	*
L. digitata = *L. ochroleuca*							0.01	0.99	
L. hyperborea > *L. ochroleuca*							2.37	0.02	*
**Phenolic content (%)**									
Species	GLS	Omni	81	2		4.85		0.09	
Detrital age × Species				2		15.24		< 0.001	***
Detrital age (*L. digitata*)				1		3.41		0.06	
Detrital age (*L. hyperborea*)				1		15.57		< 0.001	***
Detrital age (*L. ochroleuca*)				1		1.4		0.24	
L. digitata < *L. hyperborea*		Pairs					2.2	0.03	*
L. digitata = *L. ochroleuca*							0.02	0.98	
L. hyperborea > *L. ochroleuca*							2.07	0.04	*
**Carbon content (%)**									
Species	LME	Omni	81	2		4.49		0.11	
Detrital age × Species				2		9.98		0.007	**
Detrital age (*L. digitata*)				1		16.52		< 0.001	***
Detrital age (*L. hyperborea*)				1		0.04		0.85	
Detrital age (*L. ochroleuca*)				1		2.51		0.11	
**Nitrogen content (%)**									
Species	GLS	Omni	81	2		1.49		0.47	
Detrital age × Species				2		6.16		0.05	*
Detrital age (*L. digitata*)				1		13.3		< 0.001	***
Detrital age (*L. hyperborea*)				1		4.53		0.03	*
Detrital age (*L. ochroleuca*)				1		0.59		0.44	
**Carbon-nitrogen ratio**									
Species	GLM	Omni	81	2		0.54		0.77	
Detrital age				1		4.62		0.03	*

Table S7. Decomposition of Northeast Atlantic *Laminaria* species in *in situ* mesh bag experiments. Decomposition rates, given as means ± standard errors, were measured in the kelp forest at West Hoe (WH) and on sediment off Drake's Island (DI) and Jennycliff (JC) in Plymouth Sound (Fig. 2a). Depths are given in relation to lowest astronomical tide.

**Species**	**Duration**	**Site**	**Seabed**	**Depth (m)**	**Mesh ø (mm)**	**n**	**Decomposition (% d^{-1})**
L. digitata	May–Jun 2019 (32 d)	WH	Forest	2	13	27	1.01 ± 0.21
	Mar–May 2016 (40 d)	DI	Sediment	4	20	4	0.27 ± 0.12
					2	4	0.8 ± 0.15
		JC	Sediment	4	20	4	0.81 ± 0.13
					2	4	1.24 ± 0.32
L. hyperborea	May–Jun 2019 (32 d)	WH	Forest	2	13	27	0.79 ± 0.24
	Mar–May 2016 (40 d)	DI	Sediment	4	20	4	0.04 ± 0.02

					2	4	0.28 ± 0.13
		JC	Sediment	4	20	4	0.22 ± 0.09
					2	4	0.64 ± 0.47
L. ochroleuca	May–Jun 2019 (32 d)	WH	Forest	2	13	27	1.81 ± 0.34
	Mar–May 2016 (40 d)	DI	Sediment	4	20	4	0.26 ± 0.15
					2	4	0.8 ± 0.14
		JC	Sediment	4	20	4	1.42 ± 0.25
					2	4	1.82 ± 0.24

2. Figures

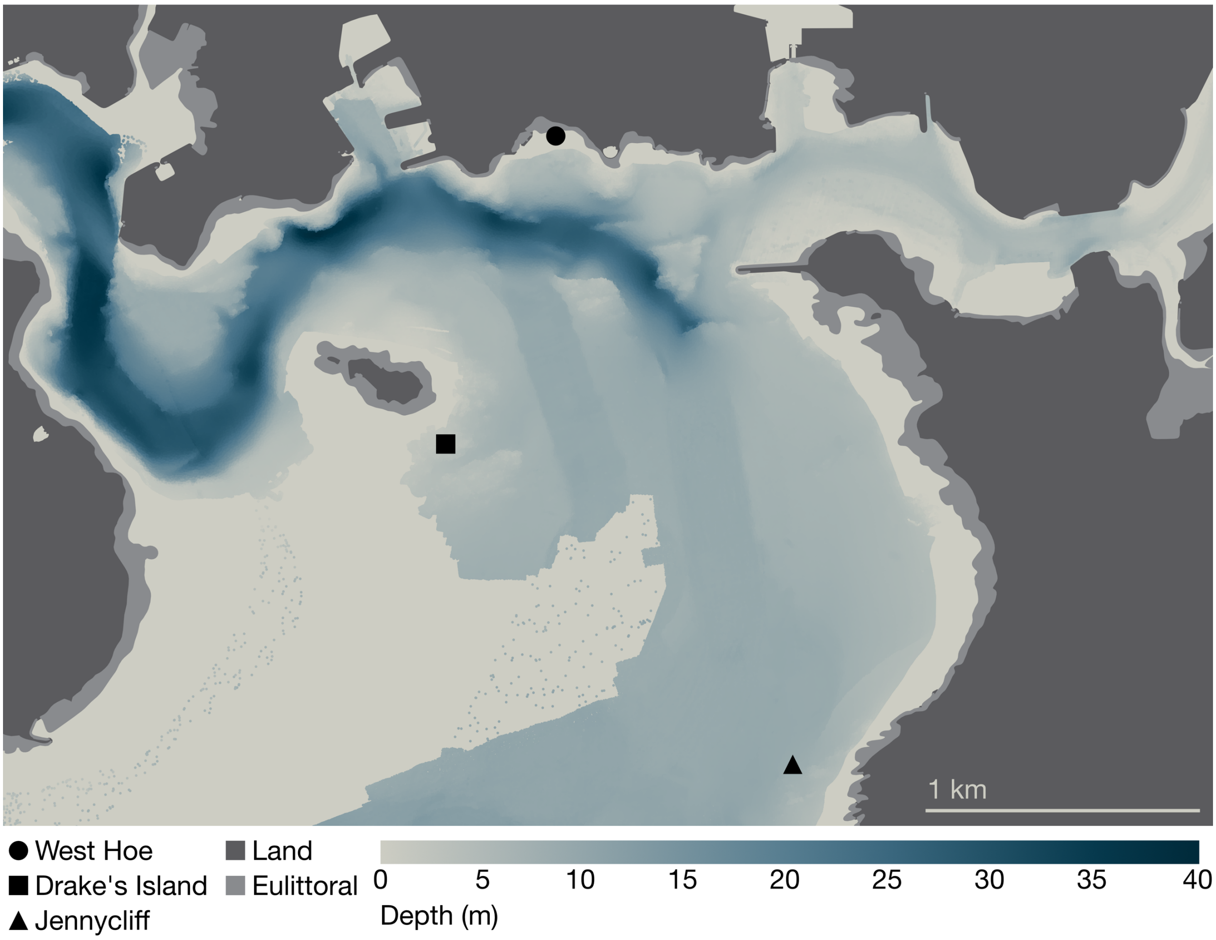

Figure S1. Juxtaposition of infralittoral kelp forest (West Hoe) and sedimentary (Drake's Island and Jennycliff) study sites in Plymouth Sound, UK, illustrating their potential connectivity. Importantly, sedimentary sites are located deeper and downstream of the kelp forest and likely receive allochthonous carbon via seaward (southward) flux of kelp detritus. The map is based on the coordinate reference system OSGB 1936, rendered according to the transverse Mercator projection and oriented north. Coastline and bathymetry data were obtained from the Ordnance Survey[79] and UK Hydrographic Office[80] respectively and rendered in QGIS v3.2 (qgis.org).

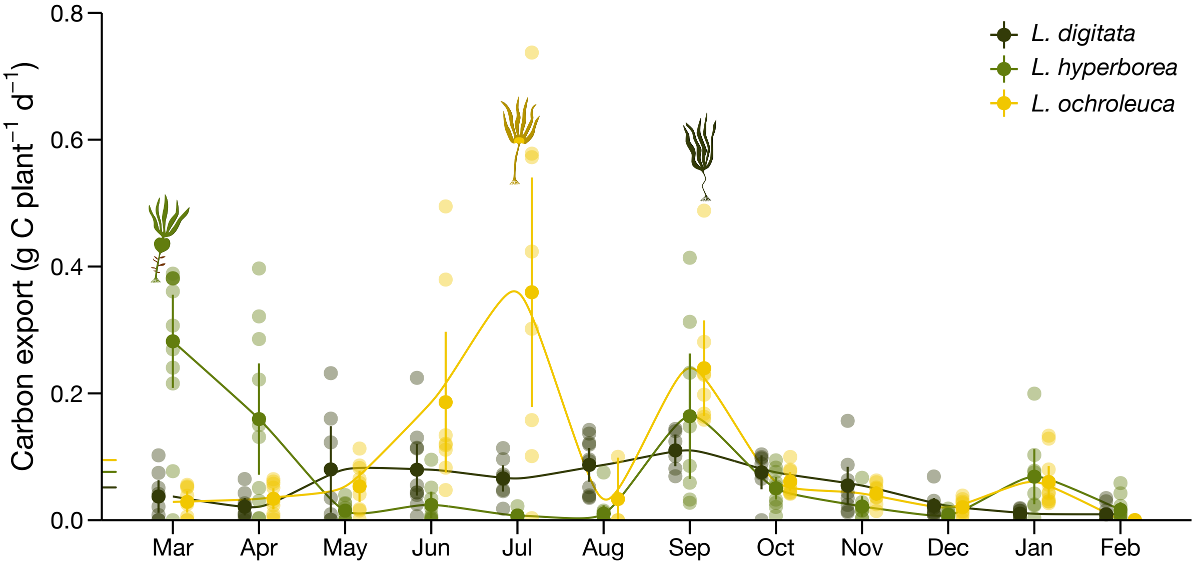

Figure S2. Seasonal particulate carbon export via distal frond erosion from Northeast Atlantic *Laminaria* species. Measurements were taken between March 2016 and February 2017 at West Hoe, the same kelp forest where *in situ* decomposition experiments took place in 2019. Point-ranges indicate means and 95% confidence intervals. Coloured axis ticks are annual means of daily carbon export. Kelp icons denote peaks in carbon export for each species.

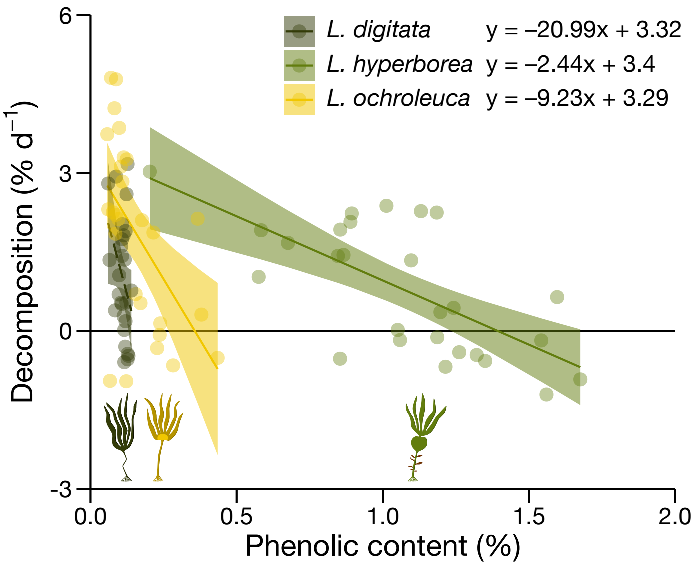

Figure S3. Relationship between phenolic content and decomposition rate in detritus of Northeast Atlantic *Laminaria* species. Lines and shaded areas are model predictions and 95% confidence intervals. Solid lines represent significant slopes at the 95% confidence level, while dashed lines indicate no significant change over time.

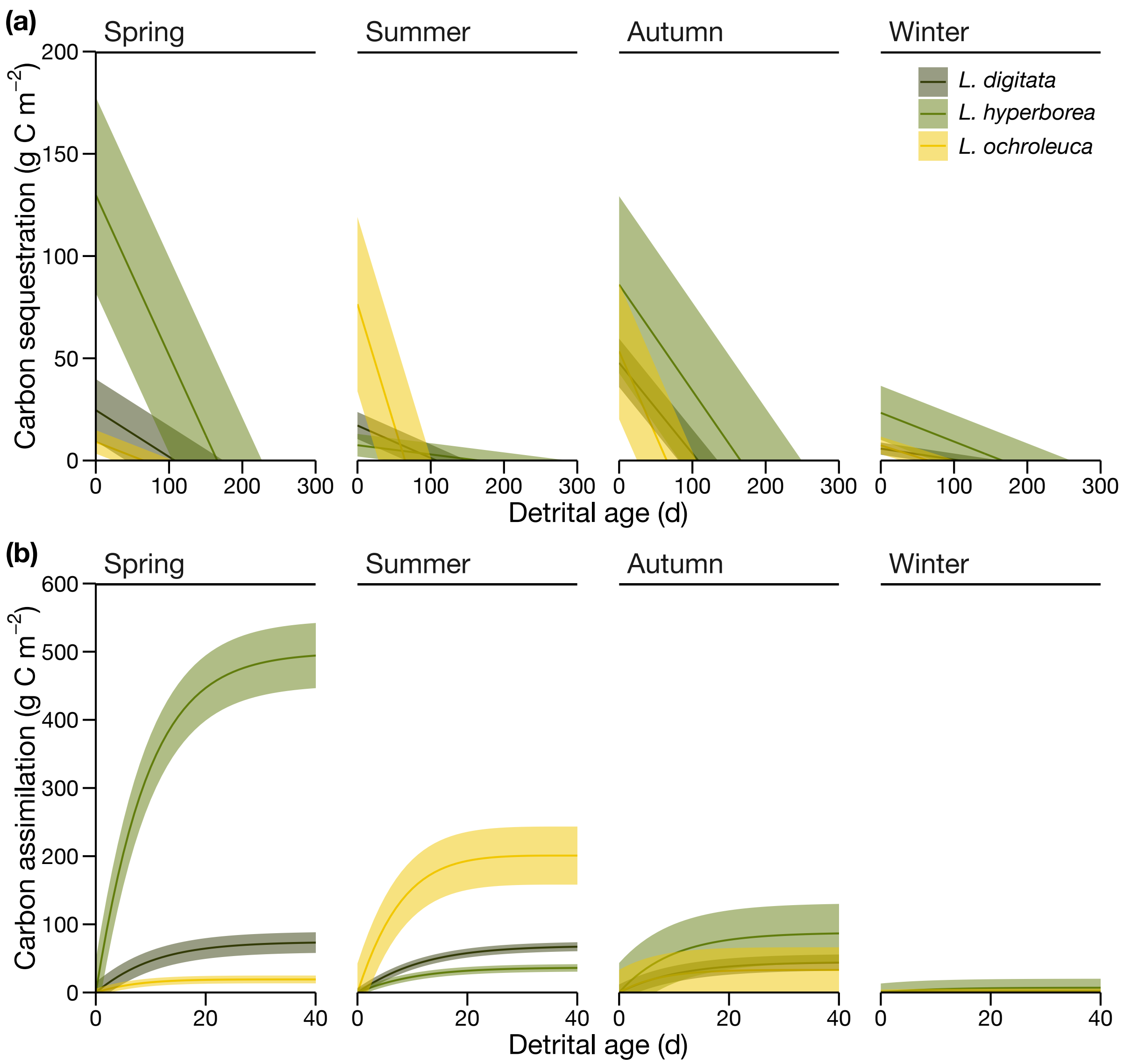

Figure S4. Present seasonal carbon sequestration potential (**a**) and cumulative net carbon assimilation (**b**) of Northeast Atlantic *Laminaria* species with increasing detrital age. Lines and shaded areas are estimates and 95% confidence intervals.

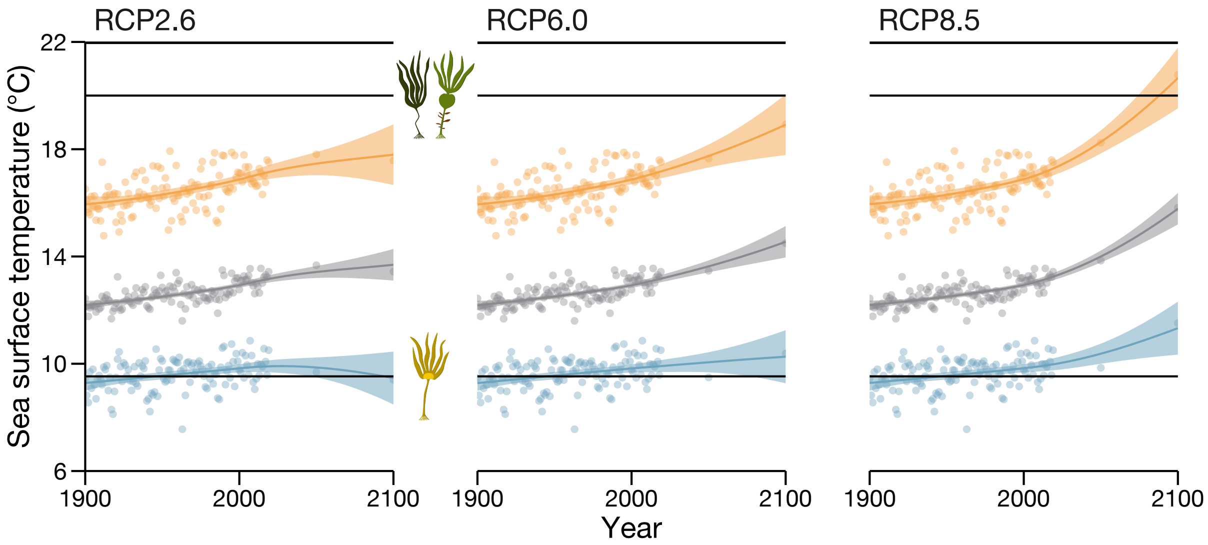

Figure S5. Historical minimum (winter, blue), mean (annual average, grey) and maximum (summer, orange) sea surface temperature data and representative concentration pathway (RCP) mid- and end-of-century predictions for the Plymouth Sound region. Lines and shaded areas are locally estimated scatterplot smoothing (polynomial regression) predictions and 95% confidence intervals. Upper and lower horizontal lines and kelp icons indicate maximum temperature tolerance for cold temperate sporophytes and minimum temperature tolerance for *Laminaria ochroleuca* gametophytes (Table S4). Note that the latter was estimated to be below the lowest tolerance of gametophyte fertility given in Table S4 (10°C) since *L. ochroleuca* was already present in Plymouth Sound in 1946, when mean winter temperatures were still approximately 9.53°C.

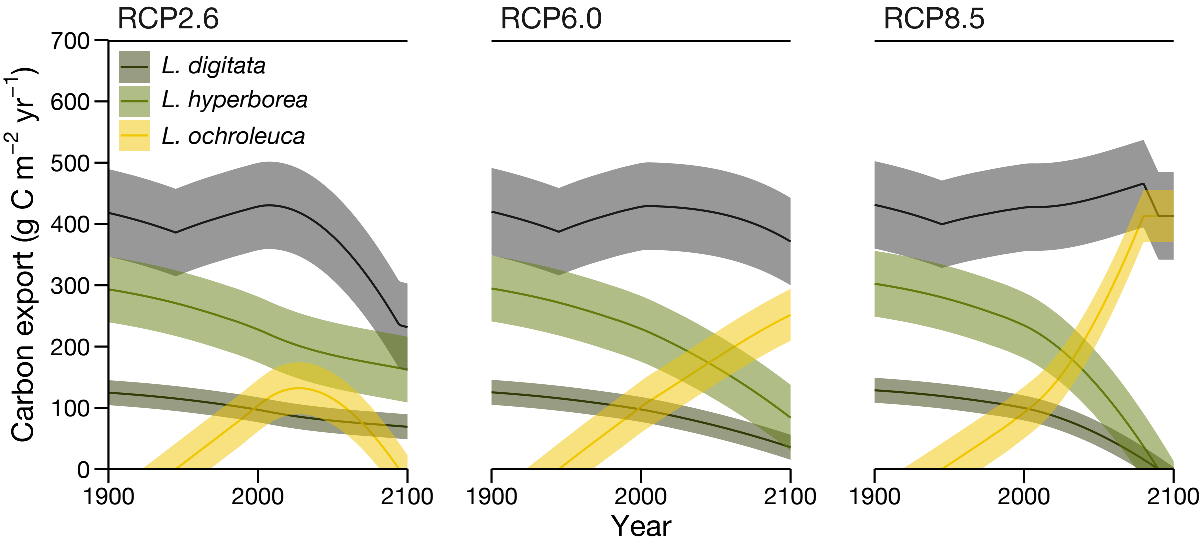

Figure S6. Temporal trend of species-specific (coloured) and overall (black) Northeast Atlantic *Laminaria* species particulate carbon export over two centuries according to historical sea surface temperature data and RCP temperature predictions for the Plymouth Sound region. Lines and shaded areas are estimates and 95% confidence intervals.

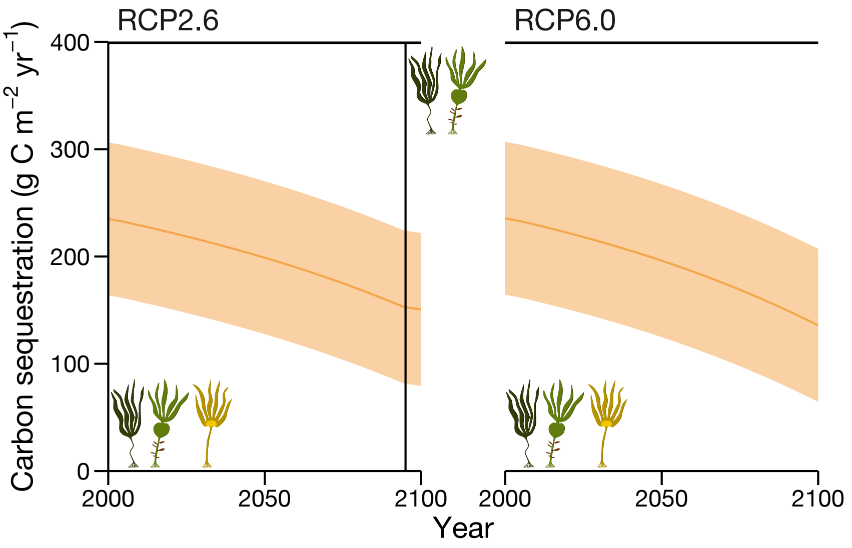

Figure S7. Temporal trend of overall kelp forest carbon sequestration potential (CSP) over one century according to RCP sea surface temperature predictions for the Plymouth Sound region. All detrital carbon remaining after 50 d (cf. Fig. 4a) is assumed to be sequestered for the purposes of highlighting the effect of changing kelp forest composition. Lines and shaded areas are estimates and 95% confidence intervals. Kelp icons and vertical lines indicate stages of compositional change.

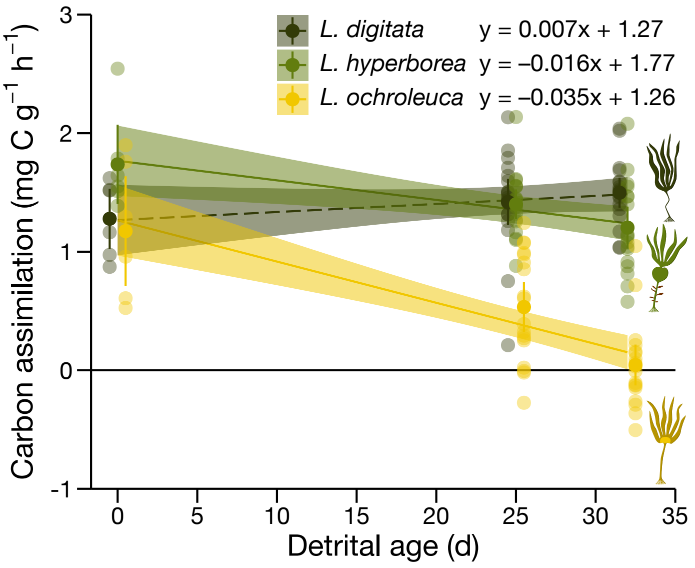

Figure S8. Effect of decomposition on gross primary production of Northeast Atlantic *Laminaria* species detritus at 50.4 μmol photons m^{-2} s^{-1}. Point-ranges indicate means and 95% confidence intervals. Lines and shaded areas are model predictions and 95% confidence intervals. Solid lines represent significant slopes at the 95% confidence level, while dashed lines indicate no significant change over time. Carbon assimilation was calculated from oxygen production, assuming photosynthetic and respiratory quotients of 1, and is given per g of dry mass.

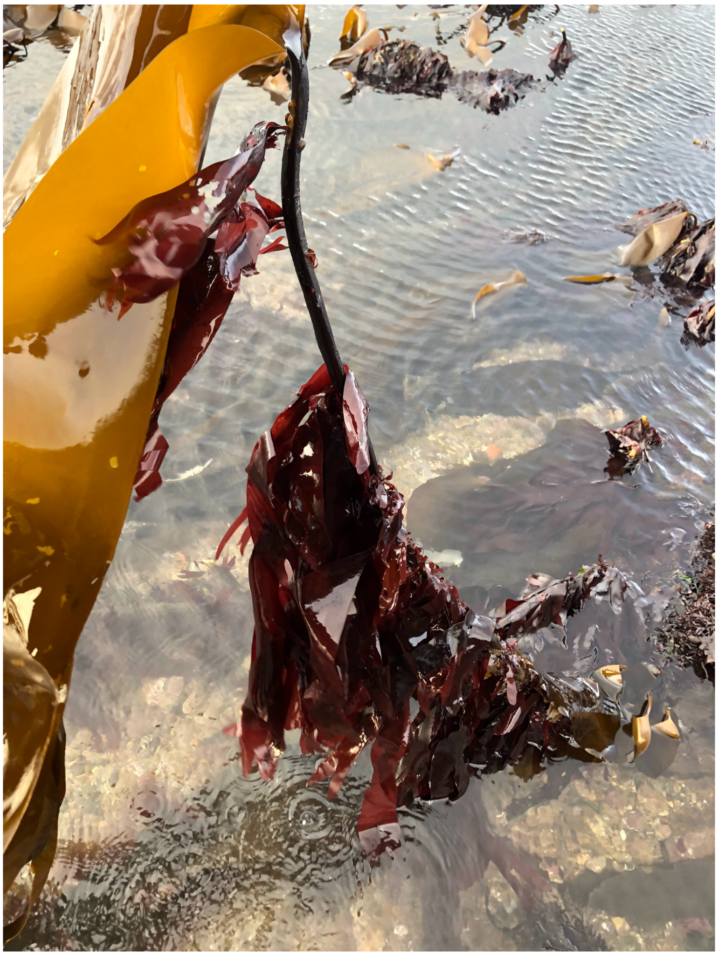

Figure S9. Mature *Laminaria digitata* sporophyte at the Walter Rocks kelp forest (54.384239°N, 5.557793°W) in Strangford Lough, UK. This photograph disproves the commonly held notion that *L. digitata* is mostly depauperate of epiphytes. © Luka Seamus Wright.

References

1. Duarte, C. M., Losada, I. J., Hendriks, I. E., Mazarrasa, I. & Marbà, N. The role of coastal plant communities for climate change mitigation and adaptation. *Nat. Clim. Chang.* **3**, 961–968 (2013).
2. Duarte, C. M. & Krause-Jensen, D. Export from seagrass meadows contributes to marine carbon sequestration. *Front. Mar. Sci.* **4**, 13 (2017).
3. Krause-Jensen, D. & Duarte, C. M. Substantial role of macroalgae in marine carbon sequestration. *Nat. Geosci.* **9**, 737–742 (2016).
4. Buitenhuis, E. T., Hashioka, T. & Quéré, C. L. Combined constraints on global ocean primary production using observations and models. *Global Biogeochem. Cycles* **27**, 847–858 (2013).
5. Ciais, P. et al. in *Climate Change 2013: The Physical Science Basis. Contribution of Working Group I to the Fifth Assessment Report of the Intergovernmental Panel on Climate Change* (eds Stocker, T. F. et al.) 465–570 (Cambridge University Press, Cambridge, 2013).
6. Middelburg, J. J. *Marine carbon biogeochemistry: a primer for Earth system scientists* (Springer, Cham, 2019).
7. Horton, T. et al. World Register of Marine Species. https://www.marinespecies.org. (2021).
8. Cebrián, J. & Duarte, C. M. The dependence of herbivory on growth rate in natural plant communities. *Funct. Ecol.* **8**, 518–525 (1994).
9. Khailov, K. M. & Burlakova, Z. P. Release of dissolved organic matter by marine seaweeds and distribution of their total organic production to inshore communities. *Limnol. Oceanogr.* **14**, 521–527 (1969).
10. Charpy-Roubaud, C. & Sournia, A. The comparative estimation of phytoplanktonic, microphytobenthic and macrophytobenthic primary production in the oceans. *Mar. Microb. Food Webs* **4**, 31–57 (1990).

11. Blinks, L. R. Photosynthesis and productivity of littoral marine algae. *J. Mar. Res.* **14**, 363–373 (1955).
12. Littler, M. M. & Murray, S. N. The primary productivity of marine macrophytes from a rocky intertidal community. *Mar. Biol.* **27**, 131–135 (1974).
13. Hillis-Clinvaux, L. Productivity of the coral reef alga *Halimeda* (order Siphonales). *Proc. Int. Coral Reef Symp.* **1**, 35–42 (1974).
14. Vooren, C. M. Photosynthetic rates of benthic algae from the deep coral reef of Curacao. *Aquat. Bot.* **10**, 143–159 (1981).
15. Littler, M. M. The population and community structure of Hawaiian fringing-reef crustose Corallinaceae (Rhodophyta, Cryptonemiales). *J. Exp. Mar. Biol. Ecol.* **11**, 103–120 (1973).
16. Littler, M. M. The productivity of Hawaiian finging-reef crustose Corallinaceae and an experimental evaluation of production methodology. *Limnol. Oceanogr.* **18**, 946–952 (1973).
17. Mann, K. H. Ecological energetics of the sea-weed zone in a marine bay on the Atlantic coast of Canada. II. Productivity of the seaweeds. *Mar. Biol.* **14**, 199–209 (1972).
18. Mann, K. H. Seaweeds: their productivity and strategy for growth. *Science* **182**, 975–981 (1973).
19. Field, P., NG, D., GS, G. & CL, V. Sun, waves, seaweed and lobsters: the dynamics of a west coast kelp-bed. *S. Afr. J. Sci.* **73**, 7–10 (1977).
20. Wanders, J. B. W. The role of benthic algae in the shallow reef of Curacao (Netherlands Antilles). I: Primary productivity in the coral reef. *Aquat. Bot.* **2**, 235–270 (1976).
21. Towle, D. W. & Pearse, J. S. Production of the giant kelp, Macrocystis, estimated by in situ incorporation of 14C in polyethylene bags 1. *Limnol. Oceanogr.* **18**, 155–159 (1973).
22. Marsh Jr, J. A. Primary productivity of reef-building calcareous red algae. *Ecology* **51**, 255–263 (1970).
23. Hawkins, C. M. & Lewis, J. B. Benthic primary production on a fringing coral reef in Barbados, West Indies. *Aquat. Bot.* **12**, 355–363 (1982).
24. Johnston, C. S., Jones, R. G. & Hunt, R. D. A seasonal carbon budget for a laminarian population in a Scottish sea-loch. *Helgol. Mar. Res.* **30**, 527–545 (1977).
25. Westlake, D. F. Comparisons of plant productivity. *Biol. Rev.* **38**, 385–425 (1963).
26. Wanders, J. B. W. The role of benthic algae in the shallow reef of Curacao (Netherlands Antilles) II: Primary productivity of the Sargassum beds on the North-East coast submarine plateau. *Aquat. Bot.* **2**, 327–335 (1976).
27. Morrissey, J. Primary productivity of coral reef benthic macroalgae. *Proc. Int. Coral Reef Symp.* **5**, 77–82 (1985).
28. Duarte, C. M. & Cebrián, J. The fate of marine autotrophic production. *Limnol. Oceanogr.* **41**, 1758–1766 (1996).
29. Kilar, J. A. & Norris, J. N. Composition, export, and import of drift vegetation on a tropical, plant-dominated, fringing-reef platform (Caribbean Panama). *Coral Reefs* **7**, 93–103 (1988).
30. Ormond, R. F. G. & Banaimoon, S. A. Ecology of intertidal macroalgal assemblages on the Hadramout coast of southern Yemen, an area of seasonal upwelling. *Mar. Ecol. Prog. Ser.* **105**, 105–105 (1994).
31. Birch, P. B., Gabrielson, J. O. & Hamel, K. S. Decomposition of Cladophora. I. Field studies in the Peel-Harvey estuarine system, Western Australia. *Bot. Mar.* **26**, 165–172 (1983).
32. Josselyn, M. N. et al. Composition, export and faunal utilization of drift vegetation in the Salt River submarine canyon. *Estuar. Coast. Shelf Sci.* **17**, 447–465 (1983).
33. Koop, K., Newell, R. C. & Lucas, M. I. Biodegradation and carbon flow based on kelp (Ecklonia maxima) debris in a sandy beach microcosm. *Mar. Ecol. Prog. Ser.* 315–326 (1982).
34. Gerard, V. A. Some aspects of material dynamics and energy flow in a kelp forest in Monterey Bay, California. https://escholarship.org/uc/item/028959vr (1976).
35. Barrón, C., Apostolaki, E. T. & Duarte, C. M. Dissolved organic carbon fluxes by seagrass meadows and macroalgal beds. *Front. Mar. Sci.* **1**, 42 (2014).
36. Rowe, G. T. & Staresinic, N. Sources of organic matter to the deep-sea benthos. *Ambio Spec. Rep.* **6**, 19–23 (1979).
37. Hardison, A. K., Canuel, E. A., Anderson, I. C. & Veuger, B. Fate of macroalgae in benthic systems: carbon and nitrogen cycling within the microbial community. *Mar. Ecol. Prog. Ser.* **414**, 41–55 (2010).

38. Lawson, G. W. & John, D. M. The marine flora of the Cap Blanc peninsula: its distribution and affinities. *Bot. J. Linn. Soc.* **75**, 99–118 (1977).
39. Assis, J. et al. Past climate changes and strong oceanographic barriers structured low-latitude genetic relics for the golden kelp *Laminaria ochroleuca*. *J. Biogeogr.* **45**, 2326–2336 (2018).
40. Bartsch, I. et al. Changes in kelp forest biomass and depth distribution in Kongsfjorden, Svalbard, between 1996–1998 and 2012–2014 reflect Arctic warming. *Polar Biol.* **39**, 2021–2036 (2016).
41. Hop, H., Wiencke, C., Vögele, B. & Kovaltchouk, N. A. Species composition, zonation, and biomass of marine benthic macroalgae in Kongsfjorden, Svalbard. *Bot. Mar.* **55**, 399–414 (2012).
42. OBIS. *Laminaria digitata* (Hudson) J.V. Lamouroux, 1813. https://obis.org/taxon/145724 (2015).
43. GBIF. *Laminaria digitata* (Huds.) J.V.Lamour. https://www.gbif.org/species/5422479 (2019).
44. NBN. *Laminaria digitata* (Hudson) J.V.Lamouroux, 1813. https://species.nbnatlas.org/species/NHMSYS0021058676 (2017).
45. van den Hoek, C. & Donze, M. Algal phytogeography of the European Atlantic coasts. *Blumea* **15**, 63–85 (1967).
46. Lüning, K. *Seaweeds: their environment, biogeography, and ecophysiology* (John Wiley & Sons, Inc., Hoboken, 1990).
47. Raybaud, V. et al. Decline in kelp in West Europe and climate. *PLoS One* **8**, e66044 (2013).
48. Assis, J., Araújo, M. B. & Serrão, E. A. Projected climate changes threaten ancient refugia of kelp forests in the North Atlantic. *Glob. Chang. Biol.* **24**, e55–e66 (2018).
49. OBIS. *Laminaria hyperborea* (Gunnerus) Foslie, 1884. https://obis.org/taxon/145725 (2014).
50. GBIF. *Laminaria hyperborea* (Gunnerus) Foslie. https://www.gbif.org/species/5422383 (2019).
51. NBN. *Laminaria hyperborea* (Gunnerus) Foslie, 1884. https://species.nbnatlas.org/species/NHMSYS0021058677 (2017).
52. Assis, J. et al. Findkelp, a GIS-based community participation project to assess Portuguese kelp conservation status. *J. Coast. Res.* **56**, 1469–1473 (2009).
53. Casado-Amezúa, P. et al. Distributional shifts of canopy-forming seaweeds from the Atlantic coast of Southern Europe. *Biodivers. Conserv.* **28**, 1151–1172 (2019).
54. GBIF. Occurence: 8 August 1960, *Laminaria hyperborea* (Gunnerus) Foslie, collected in Portugal. https://www.gbif.org/occurrence/462538635 (2019).
55. Assis, J., Lucas, A. V., Bárbara, I. & Serrão, E. Á. Future climate change is predicted to shift long-term persistence zones in the cold-temperate kelp *Laminaria hyperborea*. *Mar. Environ. Res.* **113**, 174–182 (2016).
56. OBIS. *Laminaria ochroleuca* Bachelot de la Pylaie, 1824. https://obis.org/taxon/145728 (2009).
57. GBIF. *Laminaria ochroleuca* Bachelot de la Pylaie, 1824. https://www.gbif.org/species/5422378 (2017).
58. NBN. *Laminaria ochroleuca* Bachelot de la Pylaie, 1824. https://species.nbnatlas.org/species/NHMSYS0021058678 (2017).
59. Schoenrock, K. M., O'Callaghan, T., O'Callaghan, R. & Krueger-Hadfield, S. A. First record of *Laminaria ochroleuca* Bachelot de la Pylaie in Ireland in Béal an Mhuirthead, county Mayo. *Mar. Biodivers. Rec.* **12**, 9 (2019).
60. Voerman, S. E., Llera, E. & Rico, J. M. Climate driven changes in subtidal kelp forest communities in NW Spain. *Mar. Environ. Res.* **90**, 119–127 (2013).
61. Giaccone, G. Note sistematiche ed osservazioni fitosociologiche sulle Laminariales del Mediterraneo Occidentale. *G. Bot. Ital.* **103**, 457–474 (1969).
62. Parke, M. *Laminaria ochroleuca* de la Pylaie growing on the coast of Britain. *Nature* **162**, 295–296 (1948).
63. Franco, J. N. et al. The 'golden kelp' *Laminaria ochroleuca* under global change: integrating multiple eco-physiological responses with species distribution models. *J. Ecol.* **106**, 47–58 (2018).
64. Forbes, E. in *The physical atlas: a series of maps and notes illustrating the geographical distribution of natural phenomena* (ed Johnston, A. K.) 99–101 (William Blackwood & Sons, Edinburgh, 1848).
65. tom Dieck, I. Temperature tolerance and survival in darkness of kelp gametophytes (Laminariales, Phaeophyta): ecological and biogeographical implications. *Mar. Ecol. Prog. Ser.* **100**, 253–253 (1993).

66. Bartsch, I., Vogt, J., Pehlke, C. & Hanelt, D. Prevailing sea surface temperatures inhibit summer reproduction of the kelp *Laminaria digitata* at Helgoland (North Sea). *J. Phycol.* **49**, 1061–1073 (2013).
67. Bolton, J. J. & Lüning, K. Optimal growth and maximal survival temperatures of Atlantic *Laminaria* species (Phaeophyta) in culture. *Mar. Biol.* **66**, 89–94 (1982).
68. Lüning, K. Temperature tolerance and biogeography of seaweeds: the marine algal flora of Helgoland (North Sea) as an example. *Helgol. Mar. Res.* **38**, 305 (1984).
69. tom Dieck, I. North Pacific and North Atlantic digitate *Laminaria* species (Phaeophyta): hybridization experiments and temperature responses. *Phycologia* **31**, 147–163 (1992).
70. van den Hoek, C. The distribution of benthic marine algae in relation to the temperature regulation of their life histories. *Biol. J. Linn. Soc.* **18**, 81–144 (1982).
71. Lüning, K. Critical levels of light and temperature regulating the gametogenesis of three *Laminaria* species (Phaeophyceae). *J. Phycol.* **16**, 1–15 (1980).
72. Martins, N., Tanttu, H., Pearson, G. A., Serrão, E. A. & Bartsch, I. Interactions of daylength, temperature and nutrients affect thresholds for life stage transitions in the kelp *Laminaria digitata* (Phaeophyceae). *Bot. Mar.* **60**, 109–121 (2017).
73. Müller, R., Wiencke, C. & Bischof, K. Interactive effects of UV radiation and temperature on microstages of Laminariales (Phaeophyceae) from the Arctic and North Sea. *Clim. Res.* **37**, 203–213 (2008).
74. Sjøtun, K. & Schoschina, E. V. Gametophytic development of *Laminaria* spp. (Laminariales, Phaeophyta) at low temperature. *Phycologia* **41**, 147–152 (2002).
75. Lüning, K. New frond formation in *Laminaria hyperborea* (Phaeophyta): a photoperiodic response. *Eur. J. Phycol.* **21**, 269–273 (1986).
76. Biskup, S., Bertocci, I., Arenas, F. & Tuya, F. Functional responses of juvenile kelps, *Laminaria ochroleuca* and *Saccorhiza polyschides*, to increasing temperatures. *Aquat. Bot.* **113**, 117–122 (2014).
77. Izquierdo, J., Pérez-Ruzafa, I. M. & Gallardo, T. Effect of temperature and photon fluence rate on gametophytes and young sporophytes of *Laminaria ochroleuca* Pylaie. *Helgol. Mar. Res.* **55**, 285–292 (2002).
78. Pereira, T. R. et al. Temperature effects on the microscopic haploid stage development of *Laminaria ochroleuca* and *Sacchoriza polyschides*, kelps with contrasting life histories. *Cah. Biol. Mar.* **52**, 395 (2011).
79. Ordnance Survey. OS OpenData. https://www.ordnancesurvey.co.uk/opendatadownload/products.html. (2020).
80. UK Hydrographic Office. ADMIRALTY marine data portal. https://data.admiralty.co.uk/portal/apps/sites/#/marine-data-portal. (2020).
